# Emerging Role of Calycosin in Inflammatory Diseases: Molecular Mechanisms and Potential Therapeutic Applications

**DOI:** 10.3390/biom15121643

**Published:** 2025-11-22

**Authors:** Tongzhan Liu, Yifei Ye, Yu Hu, Meixiu Jiang

**Affiliations:** 1 The Queen Mary School, Jiangxi Medical College, Nanchang University, 999 Xuefu Road, Nanchang 330031, China; 4217122148@email.ncu.edu.cn; 2 School of Pharmacy, Jiangxi Medical College, Nanchang University, 999 Xuefu Road, Nanchang 330031, China; 406700230056@email.ncu.edu.cn; 3 Jiangxi Province Key Laboratory of Bioengineering Drugs, The National Engineering Research Center for Bioengineering Drugs and the Technologies, Institute of Translational Medicine, Jiangxi Medical College, Nanchang University, 999 Xuefu Road, Nanchang 330031, China; 407400230007@email.ncu.edu.cn

**Keywords:** calycosin, Chinese herbal medicine, inflammatory diseases, anti-inflammation, anti-oxidation, therapeutic potential

## Abstract

Inflammatory diseases are a type of disease caused by multiple factors, which are characterized by local or systemic tissue inflammatory reactions, commonly including atherosclerosis, osteoarthritis, non-alcoholic fatty liver, chronic kidney diseases, acute pancreatitis, and tumors. The prevalence of the above diseases is globally high and a growing threat to human health, as well as a huge healthcare burden. In recent years, Chinese herbal medicines have become an important reservoir for the discovery of new drugs and applications due to their unique molecular structures and potential biotherapeutic effects. Numerous studies have confirmed the beneficial effects of natural products in the prevention and treatment of different diseases. Scientific studies on the therapeutic potential of natural products have become a hot topic nowadays, especially regarding the active ingredients of herbs. Calycosin is a kind of isoflavonoid extracted from the root of *Radix astragali*, exhibiting anti-inflammatory, antioxidant, anti-cancer, cardioprotective, hepatoprotective, and neuroprotective activities. Therefore, this review aims to discuss the emerging roles, molecular mechanisms and therapeutic potential of calycosin in resolving inflammatory diseases.

## 1. Introduction

Inflammation is a complex biological response set off by injuries, microbial infection, or exposure to harmful chemicals. It serves as a protective mechanism aimed at eliminating the source of injury or infection and initiating tissue repair [1]. Inflammation is characterized by the up-regulation of pro-inflammatory cytokines, such as tumor necrosis factor α (TNF-α), interleukin 1β (IL-1β), and interleukin 6 (IL-6), along with inflammatory mediators like prostaglandin E2 (PGE2) and nitric oxide (NO) released by macrophages after stimulation [2]. The infiltration of leukocytes occurs most commonly at the inflammatory phase, including neutrophils and monocytes [3]. Inflammatory responses usually exhibit characteristics of redness, swelling and pain [4]. If not treated in time, inflammation responses may result in significant tissue destruction and fuel a spectrum of inflammatory diseases, including atherosclerosis, heart failure, osteoarthritis, rheumatoid arthritis, pancreatitis, liver diseases, inflammatory bowel disease, kidney injuries, dermatitis, and even tumorigenesis, thereby posing a profound threat to human health and markedly eroding quality of life [1,5,6]. At present, steroidal and non-steroidal anti-inflammatory drugs (NSAIDs) are extensively used as a treatment strategy for most inflammatory diseases. However, with numerous obvious adverse effects after the long-term usage of these drugs, like gastrointestinal and renal complications, the need for a kind of safer and more efficient drug is necessary. Meanwhile, in most central neurological system disorders and cancers, steroidal drugs and NSAIDs are not standard-of-care due to their modest efficacy and defects. Rather than replacing these drugs, calycosin supplies a steroid-free, NSAID-free anti-inflammatory strategy by suppressing NF-κB/NLRP3 signaling. Such a profile may complement conventional immunomodulators or targeted agents to mitigate inflammation-induced damage without imposing gastrointestinal, endocrine, or bleeding defects.

In recent years, many studies have underscored the potent anti-inflammatory efficacy of Chinese herbal medicines and their extracts, attributed to their distinctive structures and fewer side effects. These traditional Chinese medicines have become a focal point of current biochemical studies, especially flavonoid compounds [7,8,9,10]. About 80% of clinical traditional Chinese medicine prescriptions contain *Radix astragali (RA, Huang Qi in Chinese)*, which was listed in the national drug and food homology in 2018 as having non-toxic effects. Calycosin (CA, C_16_H_12_O_5_) is a natural isoflavonoid compound found in the Chinese herbal medicine RA, extracted from the dry root of RA [11]. It has many pharmacological properties, with anti-inflammatory, antioxidant [12], anti-cancer [13], neuroprotective [14], and cardioprotective effects [15]. Specially, its anti-inflammatory property is mainly achieved by inhibiting or attenuating the effects of pro-inflammatory cytokines [16]. Studies also highlight its potential for immunomodulation, anti-aging, anti-diabetes, and angiogenesis [17]. Additionally, calycosin shows potent antioxidative properties by reducing oxidative stress markers and increasing antioxidant enzyme activities. In recent years, this natural compound has attracted significant scientific interest due to its diverse pharmacological and biomedical properties.

This review comprehensively analyzes the mechanisms by which calycosin acts on inflammatory diseases. We mainly focused on relevant research results from 2005 to 2024. Searches were made in multiple academic databases, including PubMed, Web of Science, Scopus, and Google Scholar. The search terms included “Calycosin” and “anti-inflammation” OR “inflammatory diseases”, which were combined with the study type and language category. We prioritized mechanistic research from in vitro, in vivo, and clinical studies. The findings are synthesized to provide a clear perspective on the anti-inflammatory effects of calycosin.

In the section below, we describe the pharmacological effects and molecular mechanisms of calycosin in detail as they pertain to the treatment of inflammatory diseases, which may indicate calycosin as a strategy for the potential therapeutic treatment of inflammatory diseases.

## 2. Emerging Roles and Underlying Molecular Mechanisms of Calycosin in Inflammatory Lesions and Diseases

**Figure 1:**
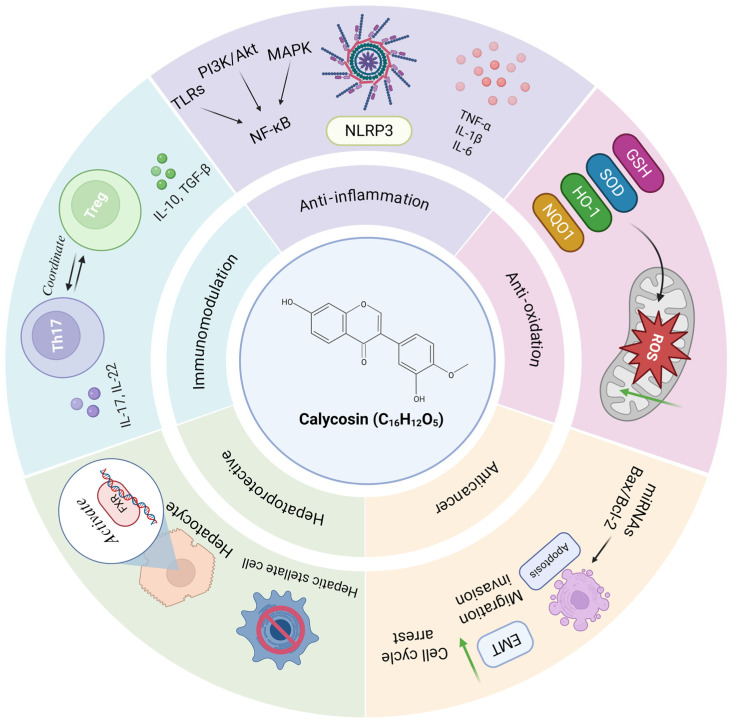
The diagram summarizes the roles and mechanisms of calycosin in inflammatory diseases. Calycosin exhibits multiple pharmacological effects, including anti-inflammatory (by inhibiting the NF-κB pathway and NLRP3 inflammasome), antioxidant (by increasing antioxidant enzyme levels and eliminating ROS), anti-cancer (by inducing apoptosis and inhibiting EMT and cell growth), hepatoprotective (by activating FXR) and immunomodulatory (by balancing Treg and Th cell function). Akt—serine/threonine kinase; EMT—epithelial–mesenchymal transition; FXR—farnesoid X receptor; GSH—glutathione; HO-1—heme oxygenase-1; IL—interleukin; MAPK—mitogen-activated protein kinase; miR—microRNA; NF-κB—nuclear factor kappa-B; NLRP3—NOD-like receptor 3; NQO1—NAD(P)H dehydrogenase quinone 1; PI3K—phosphatidylinositol three kinase; ROS—reactive oxygen species; SOD—superoxide dismutase; TGF—transforming growth factor; Th—T helper; TLR—toll-like receptor; TNF-α—tumor necrosis factor α; and Treg—Regulatory T. Green downward arrows indicate reduction.

This review systematically dissects the potential therapeutic breadth of calycosin across eight pathological arenas, including cardiovascular, articular, gastrointestinal, genitourinary, neurological, cutaneous, infectious, and neoplastic areas, while elucidating the molecular mechanisms that support its anti-inflammatory efficacy (Figure 1).

### 2.1. Cardiovascular Diseases

#### 2.1.1. Atherosclerosis

Inflammation is correlated with the progression and development of some cardiovascular diseases (CVDs). Previous studies have shown that chronic low-grade inflammation plays a significant role in the development of CVDs [18]. Atherosclerosis (AS), the common inflammatory basis of cardiovascular disease, is driven by lipid-rich intimal deposits that recruit macrophages, form foam cells, expand smooth-muscle and necrotic cores, and progressively narrow and inflame the artery [19,20,21]. Macrophages play a critical role in atherosclerosis progression, phagocytosing oxidized low-density lipoprotein (ox-LDL) to form foam cells, and thus, exacerbate plaque development. Moreover, after the stimulation of microenvironmental factors, macrophages can be polarized to different states, such as the pro-inflammatory M1 phenotype or anti-inflammatory M2 phenotype, which alternatively predominate in the inflammation progression involved in the recognition of macrophage toll-like receptors (TLRs), nuclear factor kappa-B (NF-κB) activation, and NOD-like receptor 3 (NLRP3) inflammasome assembly [22,23]. Recent research indicates that AS is not only caused by massive inflammatory responses but also unresolved inflammation [24]. Therefore, resolving inflammation and developing therapeutic drugs should be an effective strategy for the treatment of AS.

Studies have shown that calycosin can prevent AS by promoting autophagy and inhibiting foam cell formation, inflammation and apoptosis. In AS, crucial inflammatory and pro-apoptosis signaling pathways, such as the TLR-MyD88-dependent pathway, TLR-TRIF-dependent pathway, NLRP3-inflammasome pathway, Proprotein convertase subtilisin/kexin type 9 (PCSK9) pathway, Notch pathway regulating macrophage differentiation, and Wnt pathway involved in lipid deposition and inflammation initiation, are regarded as potential therapeutic targets [25]. Long-term calycosin administration at a dose of 60 mg/day·kg body weight in Apolipoprotein E gene-deficient (ApoE^−/−^) mice up-regulates the expression of krüppel-like factor 2 (KLF2) while down-regulating the expression of mixed-lineage kinase domain-like protein (MLKL) in plaque macrophages. This dual modulation drives autophagy, dampens inflammatory signaling, accelerates intracellular lipid hydrolysis, and promotes cholesterol efflux from foam cells, collectively inhibiting atherosclerotic progression [26]. Calycosin can improve perivascular adipose tissue (PVAT) dysfunction and recover its anti-contraction activity via up-regulating the adiponectin/AMP protein kinase (AMPK)/endothelial nitric oxide synthase (eNOS) pathway to produce NO and thus attenuate endothelial dysfunction and related atherosclerosis at a dose of 50 mg/kg^−1^ day^−1^ in male C57BL/6 mice [27]. In addition, 50 μM of calycosin-7-glucoside reduces Dynamin-related protein 1 (Drp1) expression and Drp1-mediated mitochondrial fission through AMPK activation in endothelial cells. The suppression of mitochondrial fission may inhibit the production of mitochondrial ROS, improve endothelial function and reduce atherosclerotic lesion formation in streptozotocin (STZ)-induced diabetic ApoE^−/−^ mice [28]. In the rat thoracic aortic smooth muscle cell line A7r5, calycosin activates the AMPK/mammalian target of the rapamycin (mTOR) pathway in a dose-dependent way at 5, 10, and 20 μM, inducing autophagy and promoting soluble N-ethylmaleimide-sensitive factor attachment protein receptor (SNARE) complex-mediated autophagosome–lysosome fusion, strengthening autophagy and mitigating vascular smooth muscle cell calcification for atherosclerosis treatment [29].

**Figure 2:**
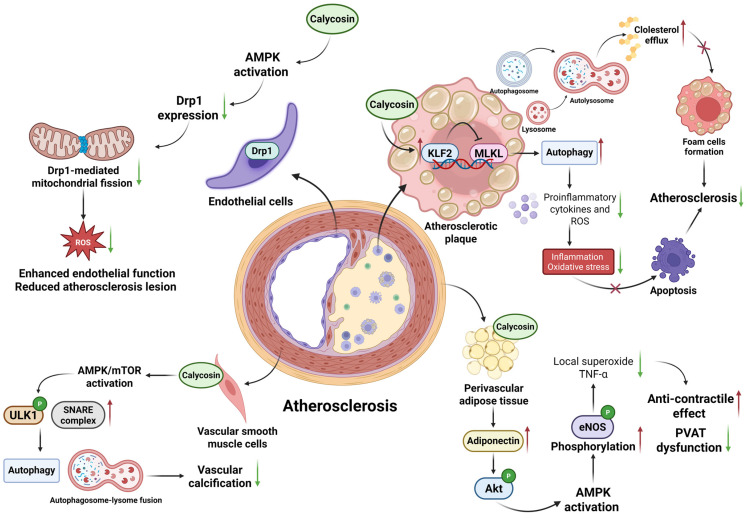
Diagram illustrating the regulated mechanisms of calycosin in atherosclerosis. Calycosin shows potential protective effects on the different layers of vessels, ranging from endothelia to perivascular adipose tissue. Calycosin prevents the progression of atherosclerosis by strengthening autophagy and cholesterol efflux to reduce cell apoptosis through the KLF2-MLKL pathway; it also enhances endothelial function, enacts an anti-contractile effect and restrains vascular calcification mainly by AMPK activation. AMPK—adenosine 5′-monophosphate (AMP)-activated protein kinase; Drp1—dynamin-related protein 1; eNOS—endothelial nitric oxide synthase; KLF2-MLKL—krüppel-like factor 2-mixed lineage kinase domain-like protein; mTOR—mammalian target of rapamycin; PVAT—perivascular adipose tissue; and SNARE—soluble N-ethylmaleimide-sensitive factor attachment protein receptor. The red upward arrows indicate increases, the green downward arrows indicate decreases, and cross marks represent inhibition.

In conclusion, calycosin can effectively mitigate inflammation and reduce lipid retention in macrophages by enhancing cholesterol efflux via regulating multiple signaling pathways, emphasizing its crucial roles in treating atherosclerosis (Figure 2). Consequently, anti-inflammatory strategies are no longer limited to conventional low-density lipoprotein synthesis inhibitor Statins and Phospholipase A2 Inhibitors [19,23].

#### 2.1.2. Heart Failure

Heart failure (HF) is a chronic cardiovascular disease related to the development of inflammation and fibrosis, usually manifesting weakness, fatigue, breathing difficulties during daily activities, limb swelling, and ascites. There are forty million people suffering from heart failure globally [30]. Patients with acute myocardial infarction [31], hypertension, heart valve disease, and myocarditis may develop HF more easily [32]. HF sufferers typically exhibit high levels of inflammatory cytokines, such as TNF-α, IL-1, IL-6 and IL-8, which amplify myocardial fibrosis and further erode the already compromised cardiac performance [33].

In proinflammatory conditioned-media (CM)-challenged H9C2 cardiomyoblasts, calycosin (5 µM) dampens inflammatory signaling and fibrotic responses by modulating the Akt-IκB kinase (IKK)/signal transducer and activator of transcription 3 (STAT3) axis. Herein, calycosin exhibits anti-inflammation effects via the phosphatidylinositol three kinase (PI3K)-serine/threonine kinase (Akt) signaling pathway, increasing the expression of PI3K and phosphorylated Akt in cardiomyocytes treated with calycosin, then down-regulating phosphorylated IκB kinase α/β and phosphorylated NF-κB to promote the survival of cardiomyocytes and attenuate inflammatory effects, as indicated by decreased pro-inflammatory cytokines TNF-α and IL-1β. Meanwhile, calycosin exhibits its antioxidative properties by reducing reactive oxygen species (ROS) levels and protecting mitochondrial function, thus preventing myocardial damage. In an in vitro TGFβ-induced cardiac fibroblast model, calycosin exhibits anti-fibrotic activity at a dose of 5 μM by decreasing α-smooth muscle actin (α-SMA) expression and reducing p-STAT3 and matrix metalloproteinase-9 (MMP-9) levels against HF [34,35]. Treatment with 50 mg/kg^−1^ day^−1^ calycosin in male C57/BL6 mice can reduce myocardial fibrosis by directly inhibiting the transforming growth factor-beta receptor 1 (TGFBR1) pathway and down-regulating intracellular signal transducers Smad2/3 to suppress the proliferation and activation of cardiac fibroblasts as well as collagen and extracellular matrix deposition against myocardial fibrosis [36]. Moreover, calycosin addresses the cardiotoxicity or HF induced by doxorubicin, a chemotherapeutic medicine, by inhibiting the activation of NLRP3 inflammasome and downstream pyroptotic caspase-1 and inhibiting membrane protein GSDMD lysis to attenuate NLRP3-mediated pyroptosis and inflammation progression at 0–20 μM in H9C2 cells in a dose-dependent way; it also increases the expression of Atg7 to promote autophagy recovery, and thus clears the damaged cellular components and attenuates cardiotoxicity at a dose of 5 µmol/L in adult zebrafish doxorubicin-induced cardiotoxicity (DIC) models [37,38]. Calycosin was found to enhance cell viability and reduce cell apoptosis in a dose-dependent way at 50–200 μM in H9c2 cells by increasing Bcl-2 expression, decreasing Bax expression and activating phosphorylated PI3K-Akt proteins to suppress oxidative stress and inflammation. It does this by up-regulating sirtuin 1 (Sirt1) expression but down-regulating NLRP3 expression [39,40]. Moreover, calycosin ameliorates triptolide-induced cardiotoxicity at a dose of 100 μM in H9c2 cardiomyocytes by promoting the formation of the peroxisome proliferator-activated receptor gamma coactivator-1 alpha (PGC-1α)/nuclear respiratory factor-1(NRF1) complex to strengthen mitochondrial biogenesis [41]. These in vitro findings suggest a potential mechanistic basis for its cardioprotective effects, although translation to in vivo heart failure outcomes remains to be established.

**Figure 3:**
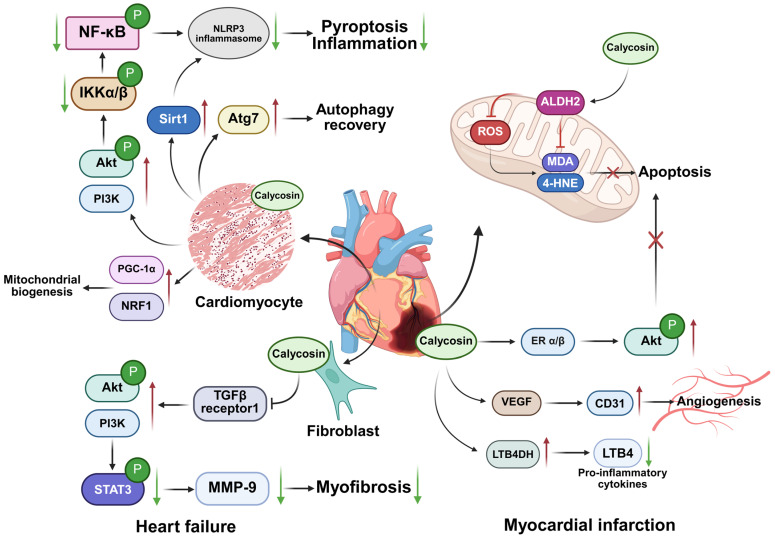
Diagrammatic representation of impacts of calycosin on heart failure and myocardial infarction. Calycosin mainly activates the PI3K/Akt signaling pathway to attenuate inflammation and pyroptosis progression and prevent myofibrosis conversion in heart failure. Furthermore, in the context of myocardial infarction, it activates ALDH2 in mitochondria to inhibit ROS production and enhance the clearance of harmful aldehydes, and it activates ERα/β to suppress cell apoptosis. Calycosin also regulates VEGF/CD31 to facilitate angiogenesis and LTB4DH/LTB4 to reduce inflammation for treatment. 4-HNE—4-Hydroxynonenal; ALDH2—aldehyde dehydrogenase 2; CD31—cluster of differentiation 31; ERα/β—estrogen receptor α/β; IKK—IκB kinase; LTB4—Leukotriene B4; LTB4DH—Leukotriene B4 12-hydroxydehydrogenase; MDA—malondialdehyde; MMP-9—matrix metalloproteinase-9; NRF1—nuclear respiratory factor-1; PGC-1α—peroxisome proliferator-activated receptor gamma coactivator-1 alpha; PI3K/Akt—phosphatidylinositol three kinase serine/threonine kinase; ROS—reactive oxygen species; Sirt1—sirtuin 1; STAT3—signal transducer and activator of transcription 3; VEGF—vascular endothelial growth factor. The red upward arrows indicate increases, the green downward arrows indicate decreases, and cross marks represent inhibition.

Overall, calycosin may be a novel therapeutic intervention for HF that assists in attenuating inflammation, reducing ROS levels and promoting autophagy to protect cardiac function (Figure 3 left), and calycosin may potentially provide a new strategy for attenuating the cardiotoxicity caused by doxorubicin.

#### 2.1.3. Myocardial Infarction

Myocardial infarction (MI) may occur as a lifelong chronic disease or a fatal disease resulting in sudden death, but without any effective treatment, about 9.5% elder people aged above 60 suffer from MI globally [42]. This condition is a main cause of death in cardiac injury and coronary artery disease patients, mainly caused by increased oxidative stress, ischemia-induced cardiac myocytes death, and apoptosis [43,44].

Calycosin exerts anti-oxidative and anti-apoptotic properties via increasing aldehyde dehydrogenase 2 (ALDH2) activity, which is responsible for scavenging harmful aldehydes such as MDA and 4-HNE to decrease cellular ROS generation, down-regulating Bax expression and up-regulating Bcl-2 expression in the concentration range of 2.5–10 µM in vitro neonatal rat cardiomyocytes, and at doses of 10–20 mg/kg^−1^ day^−1^ in and in vivo C57BL/6 mouse MI model [45]. In H9c2 myocardial cells, calycosin increases estrogen receptor α/β (ERα/β) expression and enhances PI3K/Akt phosphorylation to attenuate oxidative stress-induced cardiomyocyte apoptosis for treating ischemic cardiac injury within the concentration range of 5–20 µM [46]. Calycosin also activates vascular endothelial growth factor (VEGF) expression and increases cluster of differentiation 31 (CD31) expression at a dose of 4 mg/kg, promoting angiogenesis in the ischemic myocardium in adult male Sprague Dawley rats [47]. In the Isoproterenol-induced myocardial infarction mice model, 40 mg/kg/day calycosin and 8 mg/kg/day gallic acid can reduce neutrophil infiltration and exhibit cardioprotective effects by synergistically inducing the expression of Leukotriene B4 12-hydroxydehydrogenase (LTB4DH) and reducing neutrophil-recruiting function of leukotriene B4 (LTB4) [48]. LTB4DH is responsible for catalyzing the oxidation of pro-inflammatory cytokines LTB4 involved in inflammatory diseases [49]. Moreover, a new drug delivery system using 30 mg calycosin and 30 mg tanshinone co-loaded mitochondria, which targets the lipid–polymer hybrid nano-system by enhancing the targeted delivery of cardioprotective agents, is being applied to address the problem of insufficient drug concentrations in ischemic hearts and treat acute myocardial infarction [50,51].

Collectively, calycosin may provide a novel therapy for attenuating oxidative stress and apoptosis by modulating ALDH2, PI3K/Akt and multiple other pathways to delay MI progression (Figure 3 right), meaning treatment is no longer limited to antihypertensive drugs, anticoagulants and painkillers.

#### 2.1.4. Hypertension

Hypertension is a condition wherein the pressure of blood vessels is continuously elevated and is a high-risk factor of many cardiovascular diseases, such as myocardial infarction, heart failure, and stroke. Globally more than one quarter of people are diagnosed with this condition, and it is gradually becoming a significant source of burden on global health [52]. Increased sympathetic nervous activity, an abnormal renin–angiotensin system and an imbalance between vasoconstrictors and vasodilators are the common pathophysiological factors causing hypertension [53].

Studies have demonstrated that calycosin, as a non-competitive calcium channel blocker, can exert its vasodilation activity at a dose of 30 μmol/L, attenuating the progression of hypertension by inhibiting voltage-operated calcium channels (VOC) and receptor-operated calcium channels (ROC), respectively, in the KCl and phenylephrine (PHE)-induced contraction of male Sprague Dawley rats. Its activity is endothelium-independent and is not related to intracellular calcium release [54]. Furthermore, calycosin activates endothelial NOS/neural NOS-dependent NO production and large-conductance calcium-activated potassium channels (BK_Ca_), and enhances endothelium hyperpolarization to induce endothelium-dependent vasodilation in a dose-dependent way at 1–100 µM in human umbilical vein endothelial cells [55].

In conclusion, calycosin can lower blood pressure via its endothelium-dependent and -independent vasodilation activity, while also providing a potential natural drug for the adjuvant treatment of hypertension.

### 2.2. Joint Diseases

#### 2.2.1. Osteoarthritis

Osteoarthritis (OA) is a common chronic and inflammatory joint disease, affecting approximately 380 million adults around the world, mainly in the aging population and women, causing inconvenience in patients’ lives [56]. OA is characterized by chondrocyte apoptosis and accompanying inflammatory responses, such as the of inflammatory cytokines IL-1β. This often leads to damaged cartilage homeostasis and cartilage degradation [56,57,58]. The treatment includes pharmacological therapy for pain relief only and joint replacement, but it has the limitation of the short life of prostheses [59]. Therefore, future medical research and development is particularly crucial and urgent.

Calycosin protects against OA by targeting key signaling pathways, such as PI3K/Akt and NF-κB. Both pathways are involved in inflammation responses and chondrocyte apoptosis [60,61,62,63]. Showing a dose-dependent relationship within the concentration range of 100–400 µM, calycosin inhibits the activation of the PI3K/Akt signaling pathway caused by IL-1β, acting to reduce chondrocyte apoptosis, while calycosin inhibits the phosphorylation of p65 (a subunit of NF-κB) in IL-1β-treated chondrocytes to decrease the production of inflammatory cytokines regulated by NF-κB [64]. In addition, forkhead box O1 (FoxO1) as a regulator of cartilage formation is also improved by calycosin within the range of 1–10 µM. The above suggestions are confirmed in the model of human primary chondrocytes stimulated with IL-1β [65]. In addition, 16–64 μM calycosin can significantly improve the balance between the synthesis and degradation of cartilage displayed by increased cartilage synthesis biomarkers type II collagen (Col-2) and its transcription controller SRY-Box Transcription Factor 9 (Sox-9), and decreased cyclooxygenase-2 (COX-2) and inactivated epidermal growth factor receptor (EGFR) were also seen after calycosin treatment in in vitro ADTC5 cells [66]. In short, calycosin mainly improves OA by regulating the PI3K/Akt, NF-κB and FoxO1 pathways to inhibit inflammation and apoptosis. Calycosin may offer patients a new alternative to non-steroidal anti-inflammatory drugs (NSAIDs) and other analgesics for the treatment of osteoarthritis.

#### 2.2.2. Gouty Arthritis

Gouty arthritis (GA) is an inflammatory joint disease caused by the deposition of monosodium urate (MSU), triggering the formation and activation of inflammasomes in joints [67]. Globally, there are about fifty-three million GA events annually, mainly in men and with a continuously increasing prevalence. The only effective treatments are uric acid-lowering therapy and adjuvant anti-inflammatory drugs [68].

The levels of inflammatory cytokines, which are absent in melanoma 2 (AIM2), IL-1β and Keap1, are higher in GA than in normal conditions. Through in vitro peripheral blood mono-nuclear cells (PBMCs) and THP-1 cells, calycosin inhibits the activation of the NF-κB pathway while activating the p62 pathway at a dose of 10 μM to reduce Keap1 expression and prevent subsequent inflammatory responses caused by AIM2 inflammasomes [69], thereby protecting against GA [70]. Therefore, calycosin attenuates GA by reducing AIM2-induced inflammation via modulating NF-κB and p62-Keap1 signaling pathways.

#### 2.2.3. Rheumatoid Arthritis

Rheumatoid arthritis (RA) is a systemic autoimmune disease and common inflammatory arthritis affecting 0.5–1% of the global population, known for its effects of chronic inflammation, joint destruction, pain and stiffness, resulting in joint deformities, impaired physical function and disability due to the persistent damage of cartilage and bone in multiple joints [71,72].

In rheumatoid arthritis synovial fibroblasts, calycosin orchestrates a potent anti-inflammatory program by engaging the p62/nuclear factor-erythroid 2-related factor 2 (Nrf2) axis within the range of 10–100 μM. This drives p62 accumulation and Keap1 degradation, liberating Nrf2 for nuclear translocation and the antioxidant response elements (ARE)-driven transcription of antioxidant enzymes heme oxygenase-1 (HO-1) and NAD(P)H dehydrogenase quinone 1 (NQO1). This antioxidant surge silences pro-inflammatory cytokines and matrix metalloproteinases (MMPs), shielding cartilage from injury and enzymatic erosion [73]. Additionally, in a collagen-induced arthritis mouse model, calycosin attenuates macrophages-induced inflammatory responses in synovial tissues at a dose of 1 mg/kg by inhibiting the activation of key inflammatory pathway modulators JNK, IKKα/β, and P65, as confirmed by the decreased levels of their phosphorylated products [74]. Through molecular dynamics simulation, it was shown that calycosin can bind to Interleukin-6 receptor with high affinity, and interfere with and inhibit IL-6R to attenuate the progression of RA [75]. Therefore, the treatment of cytokines and chemokines is still an important therapeutic method against RA [76]. Calycosin treatment avoids the adverse effects of methotrexate and anti-rheumatic drugs, such as gastrointestinal and central nervous system effects and even malignancy, but further clinical applications are still required to investigate this.

**Figure 4:**
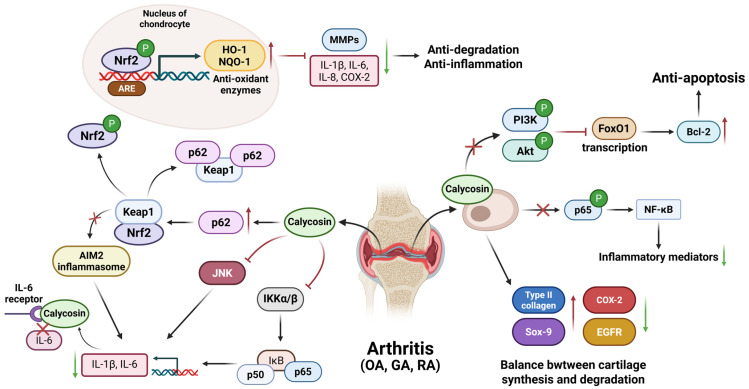
The general mechanism for calycosin-treated osteoarthritis, gouty arthritis, and rheumatoid arthritis. Calycosin in osteoarthritis interferes with cell apoptosis and inflammation, respectively, by suppressing the PI3K/Akt/FoxO1 pathway and inhibiting p65 phosphorylation. Also, it maintains the balance of cartilage synthesis and degradation by regulating type II collagen, Sox-9, COX-2, and EGFR molecules. In gouty arthritis, calycosin inhibits NF-κB while activating p62/Keap1 pathways to prevent subsequent AIM2 inflammasomes-induced inflammatory responses. In rheumatoid arthritis, calycosin mainly activates the p62/Nrf2 pathway to up-regulate antioxidant enzymes’ expression against inflammation, including HO-1 and NQO-1. It also inhibits JNK and IKK α/β to reduce the production of inflammatory cytokines, IL-1β, IL-6, etc. AIM2—absent in melanoma 2; ARE—antioxidant response element; COX-2—cyclooxygenase-2; EGFR—epidermal growth factor receptor; FoxO1—forkhead box O1; GA—gouty arthritis; HO-1—heme oxygenase-1; IKK—IκB kinase; IL—interleukin; JNK—c-Jun N-terminal kinase; Keap1—Kelch-like ECH-associated protein 1; NQO-1—NAD(P)H dehydrogenase quinone 1; Nrf2—nuclear factor-erythroid 2-related factor 2; OA—osteoarthritis; PI3K/Akt—phosphatidylinositol three kinase serine/threonine kinase; RA—rheumatoid arthritis; and Sox-9—SRY-Box Transcription Factor 9. The red upward arrows indicate increases, the green downward arrows indicate decreases, and cross marks represent inhibition.

Collectively, calycosin mitigates OA, GA, and RA mainly by regulating the p62/Nrf2, NF-κB, and other aforementioned pathways to limit oxidation stress and inflammatory cytokines release (Figure 4). Calycosin may offer patients a new alternative to treat arthritis.

### 2.3. Digestive System Diseases

#### 2.3.1. Acute Pancreatitis

Acute pancreatitis (AP) is a common acute abdominal inflammatory disease characterized by acinar cell injuries and local or systemic inflammation, such as neutrophil infiltration and releases of cytokines TNF-α, IL-6 and IL-8. With the global epidemiology of 30–90 cases per 100,000 people, some severe conditions can lead to latent multiorgan damage complications, imposing high hospitalization costs on patients [77,78]. Fluid management and nutrition are still important for AP treatment; therefore, identifying a drug that can cure AP is very important [79].

In cerulean-induced AP, calycosin treatment is effective in attenuating AP, as manifested by the decreased plasma levels of amylase and lipase. Mechanically, calycosin at a dose of 20 or 50 mg/kg inhibits the phosphorylation of IκBα and p38 mitogen-activated protein kinases (MAPK) and the subsequent expression of NF-κB/p65, leading to the reduced biosynthesis of pro-inflammatory cytokines such as TNF-α, IL-6, and IL-1β in Balb/C mice models. Furthermore, the decreased level of myeloperoxidase (MPO) and increased superoxide dismutase (SOD) activity indicate the antioxidant effects of calycosin treatment [80]. Therefore, these signaling pathways are crucial in regulating inflammation and the oxidative stress of pancreatic cells during the development of AP. The regulation of these pathways through treatment with calycosin provides a potential therapeutic option for managing the inflammatory responses and associated complications in acute pancreatitis.

#### 2.3.2. Acute Liver Failure

Acute liver failure (ALF) is a rare and severe liver disease, affecting up to one to six cases per million people and occurring commonly in patients without previous liver diseases. It is characterized by liver dysfunction, abnormally high levels of transaminases, coagulopathy, and encephalopathy, and is caused by various factors including viruses and drugs [81,82]. Cell apoptosis and systemic inflammatory responses are the main pathological features [83]. For some serious patients, emergency liver transplantation can improve their survival rates [84].

Calycosin can increase FoxM1B and SHP expression, which are the target genes of farnesoid X receptor (FXR), as well as STAT3 phosphorylation, in a dose-dependent manner, at 12.5, 25, and 50 mg/kg. This helps to reduce bile acid synthesis and promote hepatocytes proliferation and regeneration, acting against CCL_4_ induced-liver injury [85]. Further investigations into and drug development using calycosin will make it more possible to treat ALF compared to liver transplantation alone, due to the low number of suitable and paired livers.

#### 2.3.3. Non-Alcoholic Fatty Liver Disease

Non-alcoholic fatty liver disease (NAFLD) is a common chronic liver disease. The main characteristics of NAFLD progression are obvious obesity and insulin resistance. NAFLD is also related to some cardiovascular diseases, as well as type 2 diabetes mellitus, hypertension, hyperlipidemia and other hepatic metabolic syndromes [86,87]. NAFLD is divided into two types, simple hepatic steatosis and non-alcoholic steatohepatitis (NASH). Fatty liver, also known as simple hepatic steatosis, is caused by the accumulation of fats in the liver, and NASH is characterized by inflammation and fibrosis. If no treatment is applied in time, NAFLD can easily develop into liver fibrosis and cirrhosis, possibly even leading to hepatocellular carcinoma [88]. Nowadays, NAFLD has a worldwide prevalence of about 25%, with 4–8% of patients dying from liver cirrhosis. The current pharmacological options, vitamin E and pioglitazone, are hampered by safety concerns, including increased bleeding risk, prostate cancer signals and weight gain. Consequently, there is an urgent need to develop safer, more effective agents, and FXR agonists now represent one of the most promising therapeutic avenues for NAFLD [89].

To attenuate the development of NAFLD, calycosin acts as an FXR agonist to regulate the metabolism of lipids and glucoses and improves glucose tolerance and insulin resistance. FXR is a member of the nuclear receptor family, and is important for lipid metabolism and glucose homeostasis [90]. In the high fat diet-induced NAFLD model of C57BL/6J male mice, a daily oral gavage of 50 mg/kg calycosin robustly activates the FXR. Ligand-bound FXR transcriptionally up-regulates the small heterodimer partner (Shp), which, in turn, suppresses the expression of the lipogenic master regulator Sterol-regulatory element binding protein 1c (Srebp-1c) and the gluconeogenic enzymes Phosphoenolpyruvate carboxykinase (Pepck) and Glucose-6-phosphatase (G-6-pase). Consequently, de novo lipogenesis and hepatic gluconeogenesis are inhibited. Concomitantly, calycosin elevates the insulin-sensitizing proteins Glucose transporter 4 (Glut-4) and Glycogen synthase kinase 3 beta (Gsk3β), enhancing peripheral glucose uptake and glycogen synthesis, thereby ameliorating both glucose tolerance and insulin resistance [91]. Through FXR activation, calycosin also inhibits the synthesis of glyceride and increases fatty acid β-oxidation to attenuate lipid accumulation, and reduces the production of collagen I to mitigate liver fibrosis in a NASH mouse model, showing a dose-dependent relationship of 12.5, 25, and 50 mg/kg [92]. Meanwhile, calycosin can increase estrogen receptor β (ERβ) expression, and then activate the subsequent Janus kinase 2 (JAK2)-STAT3 to inhibit hepatic stellate cells’ activation and collagen deposition against carbon tetrachloride-induced liver fibrosis [93,94,95,96]. Bioinformatic and biochemical findings show that ALDH2, Niemann pick C1 (NPC1), and high mobility group protein 1 (HMGB1), which are responsible for lipid degradation and lipid homeostasis, may represent new therapeutic targets of calycosin in treatments against hepatic steatosis [97]. The early management of dietary changes and medical treatment, such as the use of calycosin, can reverse pathological changes in the liver in time rather than letting it develop into severe cirrhosis [98].

**Figure 5:**
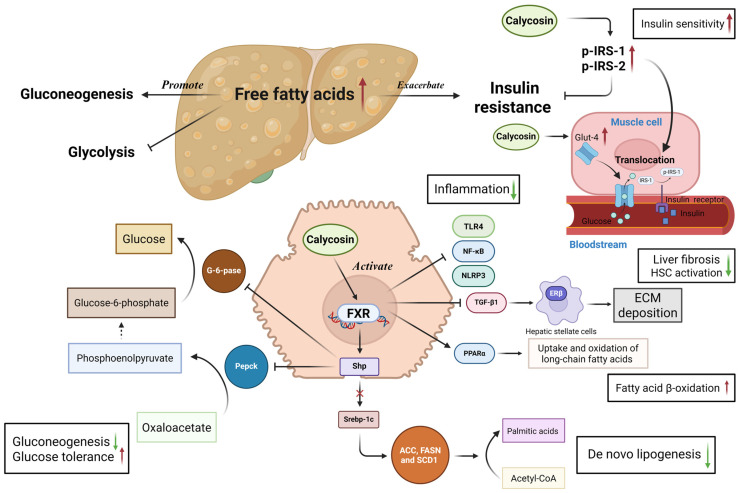
Effects and mechanisms of calycosin in the context of liver disease. Calycosin acts as an agonist of the farnesoid X receptor signaling pathway to maintain the homeostasis of glucose and lipids in the liver. Calycosin activates FXR to facilitate the expression of the small heterodimer partner, after which it inhibits sterol-regulatory element binding protein 1c, Acetyl-coenzyme A carboxylase, fatty acid synthase, and stearoyl-coenzyme A desaturase 1 expressions, which are responsible for lipogenesis. FXR activation up-regulates the expression of peroxisome proliferator-activated receptor α to increase fatty acid β-oxidation and attenuate fatty acid accumulation. Meanwhile, calycosin suppresses gluconeogenesis via the Shp-mediated inhibition of phosphoenolpyruvate carboxykinase and glucose-6-phosphatase and increases the expression of glucose transporter 4 and phosphorylase insulin receptor substrate 1/2 to promote glucose transport into cells and recover insulin sensitivity. Moreover, calycosin attenuates liver injuries and inflammation by down-regulating the TLR4-mediated nuclear factor kappa-B pathway and NOD-like receptor 3 inflammasome, reducing the risk of liver fibrosis via inhibiting hepatic stellate cell activation-induced extracellular matrix deposition. ACC—Acetyl-coenzyme A carboxylase; ECM—extracellular matrix; FASN—fatty acid synthase; FXR—farnesoid X receptor; G-6-pase—glucose-6-phosphatase; Glut-4—glucose transporter 4; HSC—hepatic stellate cells; NF-κB—nuclear factor kappa-B; NLRP3—NOD-like receptor 3; Pepck—phosphoenolpyruvate carboxykinase; p-IRS-1/2—phosphorylase insulin receptor substrate 1/2; PPARα—peroxisome proliferator-activated receptor α; SCD1—stearoyl-coenzyme A desaturase 1; Shp—small heterodimer partner; Srebp-1c—sterol-regulatory element binding protein 1c; and TLR4—toll like receptor 4. The red upward arrows indicate increases, the green downward arrows indicate decreases and the cross mark represents inhibition.

Collectively, calycosin mainly activates FXR to balance the metabolism of lipids and glucose, providing an effective medication that is not limited to anti-obesity and anti-hyperglycemia drugs alone (Figure 5).

#### 2.3.4. Diabetes Mellitus

Diabetes is a chronic metabolic disease with characteristics of hyperglycemia, polydipsia, polyuria and weight loss, commonly caused by insulin secretion deficiency (type 1) and insulin resistance (type 2). In severe circumstances, type 1 diabetes will lead to diabetic ketoacidosis and type 2 diabetes causes hyperosmolar hyperglycemia syndrome, both of which can be threatening to life [99]. Worldwide, 346 million people suffer from diabetes, according to the World Health Organization. Simple routine insulin injection is not enough to control and treat diabetes, and so researchers are studying the application of a traditional Chinese medicine for diabetes [100].

Studies have demonstrated that advanced glycation end products (AGEs) are glycosylatic and oxidative products of proteins and lipids, and are crucial in the development of diabetes, leading to inflammation, oxidative stress, cell apoptosis and insulin resistance [101]. In a normal rat hepatocyte cell line (BRL-3A) cultured by high glucose, at the optimal effective concentration of 1 × 10^−7^ M, calycosin can reverse the reduced expression of glucose transporter-1 (Glut-1) which exists in most cells and is responsible for glucose uptake, caused by AGEs being at a higher level than normal. Calycosin also binds its estrogen receptor on the cell membrane to reduce the expression of RAGE, and thus, inhibit AGEs, which also decreases the intracellular level of cAMP, and this inhibits the functions of Glut-1. Moreover, calycosin can directly bind to AGEs to inhibit their effects [102]. In human umbilical vein endothelial cells, 10^−4^ M calycosin can ameliorate AGEs-induced cell apoptosis via inhibiting AGEs–RAGE ligation and subsequently increasing anti-apoptotic protein Bcl-2 expression, while decreasing pro-apoptotic protein Bad/Bax expression to attenuate oxidative stress and vascular endothelial cells loss [103]. In addition, calycosin acts as an α-glucosidase inhibitor, inhibiting the uptake of glucose from the intestinal tracts to blood via the brush border membrane with a half maximal inhibitory concentration (IC_50_) of 6.84 ± 1.58 µM. This α-glucosidase is an enzyme that catalyzes disaccharides into monosaccharides [104]. In gestational diabetes mellitus (GDM), calycosin exerts efficient effects that suppress inflammation and strengthen beta cells’ function, aiding in treating GDM in pregnant C57BL/KsJ-Lep (db/+) diabetic mice at the doses of 15 and 30 mg/kg. This operates by down-regulating RNF38 expression, inhibiting STAT3 activation and increasing the expression of SHP-1. Herein, STAT3 is involved in insulin resistance and the progression of diabetes, as regulated by RNF38 and SHP-1 [105].

Therefore, calycosin mainly increases glucose absorption and the RAGE signaling pathway to lower the glucose in blood, helping to attenuate the progression of diabetes mellitus, thus providing a novel approach to diabetes treatment supplemented by diet improvement and moderate exercise.

#### 2.3.5. Colitis

As a type of inflammatory bowel disease, colitis is a chronic and non-infectious inflammatory disease occurring in the gastrointestinal tract, mainly the rectum and colon, usually affecting 0.5 to 31.5 people per 100,000 globally each year [106]. Colitis is characterized by mucosal and submucosal inflammation, rectal bleeding, diarrhea, abdominal pain, fever, and weight loss [107]. The etiology of colitis is complex, mainly related with genetic and immune factors and environmental influences [108]. Steroids and immunosuppressants are used to treat colitis, but with many side effects, medications directly tailored for colitis are urgently needed for application in the clinic [106].

In the context of dextran sulfate sodium (DSS)-induced colitis in mice, calycosin displays positive anti-inflammatory and anti-oxidative properties and attenuates DSS-induced damaged mucosa and the presence of infiltrated inflammatory cells in the colon. Mechanically, calycosin inhibits the activation of the MAPK/c-Jun N-terminal kinase (JNK) signaling pathway via the application of daily oral gavages of 25 and 50 mg/kg. These decrease the phosphorylation levels of IKKα/β, IκBα, and p65, leading to reduced expression levels of proinflammatory cytokines, including IL-1β, IL-6, TNF-α, inducible nitric oxide synthase (iNOS), and interferon-γ (IFN-γ). Meanwhile, calycosin alleviates DSS-induced abnormal redox reactions and antioxidative enzyme activity by increasing the concentration of glutathione (GSH) and SOD to protect against colitis [109]. Moreover, calycosin inhibits the TGF-β/Smad pathway by down-regulating p-Smad2, p-Smad3, Smad4, and TGF-β1 expression and increasing Smad7 expression in human intestinal fibroblasts (CCD-18Co) cells at concentrations of 12.5, 25, and 50 μmol/L, which helps to inhibit the intestinal fibrosis caused by local inflammation [110]. This endows us with a therapeutic agent for use against inflammatory bowel diseases that is potentially more efficient than others, except for glucocorticoids and 5-aminosalicylic acid (5-ASA) [111].

### 2.4. Urinary System Diseases

#### 2.4.1. Diabetes-Induced Renal Inflammation

Diabetes-induced renal inflammation, also termed diabetic nephropathy (DN), is a severe microvascular inflammatory complication of type 1 and 2 diabetes; only 30–40% patients with diabetes develop DN, with the cellular mechanisms of growth factors, oxidative stress, proinflammatory cytokines, and chemokines [112,113]. This usually leads to chronic kidney disease and end-stage renal disease without effective treatment [114].

Calycosin effectively alleviates renal injuries in C57BL/KsJ (db/db) mice through intraperitoneal injection with 10 mg/(kg·d) and reduces the production of TNF-α and IL-1β at doses above 10 μM in mouse tubular epithelial cells (mTEC). It inhibits diabetes-induced renal inflammation by inhibiting the phosphorylation of inhibitor of NF-κB (IκBα) to prevent NF-κB activation [115]. In addition, calycosin protects renal cells and improves cell viability in high-glucose-induced renal tubule injury by inhibiting ferroptosis within the range of 0, 5, 10, 20, 40, and 80 μM, this being an iron-dependent cell death [116]. The daily oral gavage of 5 mg/kg calycosin can inhibit the IL-33/ST2 axis and lead to the substantial activation of the NF-κB inflammatory pathway and the TGF-β/Smad pathway while also activating the Nrf2/ARE pathway to attenuate diabetes-induced inflammation, renal fibrosis and oxidative stress in high fat diet-fed/STZ-injected rats [117]. Moreover, studies suggest that calycosin can be distributed into multiple organs such as the kidneys and lungs through oral administration in murine tissues, with positive and beneficial effects in the treatment of kidney diseases [118]. Recently, a new therapeutic technique has been developed using calycosin-loaded nanoliposomes to alleviate DN by reducing oxidative stress damage and restoring mitochondrial functions in the kidney cells [119]. As a result, calycosin attenuates inflammation during DN progression, achieving an anti-inflammatory effect that assists renin–angiotensin system blocker treatment. This represents a promising avenue for research into problems for which there are currently no viable treatments.

#### 2.4.2. Renal Ischemia/Reperfusion Injury

Renal ischemia/reperfusion injury (RIRI) is an irreversible type of tissue damage mainly caused by inflammatory responses, including immune cells and the secretion of inflammatory cytokines [120]. Studies show that necrosis, apoptosis, and inflammation are involved in the progression of RIRI, and ischemia/reperfusion injury represents a big challenge in the context of kidney transplantation arising from donor kidneys being in the ischemic state and acceptors being in the proinflammatory state [121,122]. Renal IRI is also a common cause of acute kidney injury (AKI), affecting about 11% of all hospitalized people, and it lacks an effective treatment [120].

In male C57BL/6 mice with RIRI, calycosin alleviates renal injury by reducing NF-κB mediated inflammatory responses within the range of 5, 10, and 20 mg/kg. It achieves this by up-regulating the expression of peroxisome proliferator-activated receptor γ (PPARγ) and suppressing early growth response 1 (EGR1), which is a transcription factor for the release of pro-inflammatory mediators through the NF-κB pathway [123]. Therefore, Calycosin alleviates RIRI and shows its anti-inflammation effects through the PPARγ/EGR1 pathway, all while attenuating oxidative stress injuries and providing vascular protection in RIRI.

#### 2.4.3. Chronic Prostatitis

Chronic prostatitis (CP) is a common inflammatory disease occurring in the prostate gland, mainly found in middle-aged men with an estimated prevalence of 4.5–9% worldwide. It is characterized by abnormal or frequent urination, chronic pain of the pelvic or lower abdominal area, and sexual dysfunction, lowering the life quality of patients [124,125]. Meanwhile, treatments for CP, such as analgesic, physiotherapy, and prostate massage, are palliatives and are not directed at the causes of the disease itself [124]. This is the reason for the research into and development of new drug therapies for CP treatment.

Studies shows that calycosin can alleviate damage to the prostate tissues of rats with CP by inhibiting the activation of the p38 MAPK/NF-κB signaling pathway through daily oral gavages of 10, 20, and 30 mg/kg. Both inflammation and oxidative stress are crucial in the development of CP. Calycosin shows its anti-inflammatory property by inhibiting the phosphorylation level of p38 and p65 to protect against CP and reduce the production of pro-inflammatory cytokines. Meanwhile, ROS and MDA, products of lipid peroxidation, are higher in samples with CP than in those with a normal condition. Furthermore, ROS can activate the p38 MAPK/NF-κB pathway to facilitate the release of inflammatory cytokines. With calycosin treatment, the levels of ROS and malondialdehyde (MDA) are decreased [126]. Therefore, calycosin protects against CP mainly by regulating the p38 MAPK/NF-κB signaling pathway to achieve anti-inflammation and anti-oxidation, providing a novel target for the management of CP.

### 2.5. Nervous System Diseases

#### 2.5.1. Intracerebral Hemorrhage Induced Brain Damage

Intracerebral hemorrhage (ICH) is the most lethal cause of stroke, with a global prevalence of 3.4 million cases per year, and often occurs among people under the age of 50 [127]. In the cases of the most common causes, hypertension and vascular lesions, ICH commonly comprises two different injuries; primary injury refers to damage to the brain and hematoma induced by initial bleeding, while secondary injury refers to a chain of events after primary injury, such as inflammatory responses, oxidative stress and cell death [128].

To protect against brain damage and attenuate inflammation, studies have reported that calycosin exerts its neuroprotective function via blocking the activation of the NLRP3 inflammasome and the classical NF-κB pathway to reduce ICH-induced inflammation [129]. In a collagenase type VII-induced ICH mouse model subjected to calycosin treatment, the levels of antioxidant enzyme Nrf2 and Sod1 were higher, thus reducing oxidative stress and activating the NF-κB pathway and NLRP3 following the application of calycosin at a concentration of 50 mg/kg, this being an effective dose that achieves a plateau effect [130]. Although research on calycosin in the treatment of ICH is still in the early stage, the above mechanisms indicate its potential value in restoring ICH, but further studies are needed to explore this.

#### 2.5.2. Cerebral Ischemia Injury

Cerebral ischemia/reperfusion injury (CIRI) comprises the secondary pathological damage and dysfunction caused by ischemic stroke in ischemic brain tissue, mainly caused by oxidative stress, inflammation, mitochondrial dysfunction, autophagy and apoptosis [131]. Ischemic stroke is a main cause of severe disability and death among the aged, with 7.8 million cases globally every year, characterized by transient or permanent decreases in cerebral blood flow, leading to reduced oxygen and nutrition supply in the brain tissue [132,133].

Li, X et al. reported that calycosin exerts anti-inflammatory and neuroprotective effects in the treatment of CIRI via inhibiting the HMGB1/TLR4/NF-κB signaling pathway using an oxygen–glucose deprivation/reoxygenation (OGD/R) model of rat microglia, with the optimal effective concentration range of 1–4 μM, and with the finding of cytotoxicity above 100 μM [134]. In addition, calycosin can inhibit autophagy via the STAT3/FOXO3a signaling pathway at the effective dose of 30 mg/kg in a rat middle cerebral artery occlusion/reperfusion (MCAO/R) model, and alleviates special acyl-CoA synthetase long-chain family member 4 (ACSL4)-dependent ferroptosis at the concentration of 60 μM in in vitro PC12 cells, helping to alleviate CIRI [131,135]. In the same MCAO rat model, it was found that calycosin protects against CIRI within the range of 5–20 mg/kg by increasing the expression of autophagy-related protein p62 and neighbor of BRCA1 gene 1 (NBR1) and anti-apoptotic Bcl-2, producing anti-autophagic and anti-apoptotic activity [136]. Meanwhile, calycosin therapy increased brain-derived neurotrophic factor (BDNF) and tropomyosin-related kinase B (TrkB) expression in the brain at a dose of 30 mg/kg, leading to the switching of the TNF-α-containing microglia from an activated to a resting state, helping to attenuate inflammation and neurological injury [137]. Moreover, calycosin reduces Dexamethasone-induced Ras-related protein 1 (RASD1) expression, and up-regulates ER-α, microRNA (miR)-375 and Bcl-2 to reduce the infarct volume and brain water content, which is beneficial against CIRI at doses of 5, 10, and 20 mg/kg [138]. Liu, W. et al. found that the Sphingosine1-phosphate (S1P)/Sphingosine-1-phosphate receptor1 (S1PR1)/PI3K/Akt pathways are activated by the application of 0.44 mg/kg daily calycosin, helping to inhibit neuronal apoptotic death and protect against CIRI and ischemic stroke [139]. Guo, C. et al. discovered that calycosin shows anti-oxidative neuroprotective properties at the optimal effective dose of 30 mg/kg by reducing MDA, protein carbonyl and ROS, and up-regulating superoxide dismutase, catalase, and glutathione peroxidase (GSH-Px) [140]. Similarly, Guo, C. et al. reported that calycosin can inhibit calpain activation and increase transient receptor potential canonical 6 (TRPC6) and cAMP-response element binding protein (CREB) expression at the dose of 5–20 mg/kg, helping to protect neurons from oxygen glucose deprivation [141]. Zhang, W. et al. found that calycosin at the concentration of 30 μmol/L inhibits T-cell mitogen Con A-induced T cells’ proliferation, thus helping to attenuate inflammatory responses [142]. Moreover, Lu, C. stated that calycosin can activate Akt, promote the phosphorylation of Nrf2 and downstream HO-1 and SOD activation to limit H_2_O_2_-induced ROS production, and attenuate oxidative stress, all helping to protect rat brain astrocytes against ischemic injuries within the concentration range of 0–100 μM [143]. Therefore, calycosin shows extensive and potential therapeutic mechanisms in the treatment of cerebral ischemic injuries, including anti-inflammation, anti-autophagy, and anti-cell death.

### 2.6. Skin Diseases

#### 2.6.1. Allergic Dermatitis

Allergic dermatitis (AD) is a common skin disease entailing chronic inflammation and frequent reoccurrence, and is mainly caused by the dysfunction of the skin barrier, placing a considerable psychological burden on patients [144]. It is a prevalent condition, affecting up to 250 million people worldwide, with the possibility of affecting individuals at any age [145]. The avoidance of allergens is still important for curing dermatitis; meanwhile, a drug that can repair epithelial dysfunction is being sought.

To treat allergic dermatitis, calycosin down-regulates hypoxia-inducible factor (HIF)-1α to repair epithelial tight junctions, inhibits the TLR4-mediated NF-κB signaling pathway and reduces thymic stromal lymphopoietin (TSLP) production within the intraperitoneal injection dose range of 2–50 mg/kg in Balb/C mice and at the concentration of 10 μmol/L in HaCaT cells. Specifically, the tight junctions are damaged by high levels of HIF-1α, while calycosin decreases the expression of HIF-1α and improves the expression of tight junction proteins including occludin, zonula occluden-1 (ZO-1), and claudin1 (CLDN1), helping to repair the epithelium [144,146]. Similarly, calycosin can interact with GPER to increase occludin expression, improve E-cadherin distribution, and inhibit TSLP production, contributing to protecting the epithelial barrier in allergic asthma treatments at a dose of 10 mg/kg in a house dust mite (HDM)-induced allergic asthma mouse model, and at the effective concentration of 10 μM in the TNF-α- and Poly (I:C)-co-stimulated human bronchial epithelial cell line [147]. Herein, TSLP is a proallergic cytokine and a key factor in initiating allergic inflammation by inducing the activation of inflammatory cells, and it is secreted by epithelial cells. Therefore, calycosin can repair epithelial tight junctions and provide relief to allergic dermatitis.

#### 2.6.2. Atopic Dermatitis

Atopic dermatitis is a chronic inflammatory skin disease, mainly characterized by persistent itching and recurrent eczematous lesions. Inflammation and immunological modulation are involved in the progression of atopic dermatitis [148,149]. With a high prevalence of about 10–20% in developed countries, the prevalence in adults is around 10%. Topical corticosteroids are the main first-line therapy, but as these entail many adverse effects, specific disease-modifying drugs are currently being developed [150].

Calysosin alleviates epithelial disruption by inhibiting TLR4 expression within the range of 0.4–10 mg/kg, thereby inhibiting the activation of NF-κB signaling pathway [151]. With the participation of the immune system, T helper (Th) 17 cells are regarded as pro-inflammatory T helper cells. As promoters of inflammation, they can secrete inflammatory cytokine interleukin-17 (IL-17) leading to an inflammatory response. Regulatory T (Treg) cells are inhibitors of inflammation, showing immunosuppressive effects. Both of these are targets of chronic inflammation [152,153]. In a calcipotriol-induced mouse model, calycosin alleviates dermatitis by coordinating the balance of Th17 cells and Treg cells, promoting the differentiation of Treg cells but inhibiting Th17 cells at an effective local application concentration of 5 mg/mL. The subsequent inflammatory response is then also attenuated by calycosin, reflected by the decreasing levels of NF-κB-related inflammatory cytokines such as IL-1β, IL-6, and TNF-α [154]. This indicates that calycosin exerts its anti-inflammatory property, which is beneficial for treating atopic dermatitis, by modulating the relative amounts and activities of Treg and Th17 cells.

### 2.7. Infectious Diseases

#### 2.7.1. Sepsis-Induced Acute Lung Injury

Sepsis is a devastating condition, often causing multiple organ failure and leading to the high mortality rate of 30–45% in hospitalized patients. The lungs are easily infected by sepsis, and acute lung injury is induced by inflammatory damage including sepsis and pneumonia, this being characterized by acute septicemia and respiratory distress. Inflammation and oxidative stress are regarded as two crucial targets of acute lung injury treatment [155,156].

Studies have reported that calycosin can relive sepsis-induced acute lung injury (ALI) by inhibiting the HMGB1/myeloid differentiation factor 88 (MyD88)/NF-κB pathway and activating the NLRP3 inflammasome. HMGB1 is a non-histone nucleoprotein that is strongly associated with sepsis. It can transmit the signal of extracellular LPS into the cytoplasm, leading to inflammatory responses [157]. In a cecal ligation and puncture (CLP)-induced sepsis rat model, calycosin treatment was shown to decrease the levels of HMGB1, MyD88, p-NF-κB, and NLRP3, as well as the contents of pro-inflammatory cytokines such as TNF-α and IL-1β, at the significantly effective dose of 50 mg/kg [158]. Also, calycosin attenuates neutrophil infiltration and inflammation by inhibiting the HMGB1 and NF-κB pathways in L-arginine-induced acute pancreatitis-associated ALI at the doses of 25 and 50 mg/kg [159]. Moreover, calycosin has shown improved outcomes in alleviating sepsis-induced acute lung injury by targeting the mitochondrial ROS-mediated inflammasome, at doses of 12.5, 25, and 50 mg/kg, in vivo. Treatment with calycosin not only inhibits the production of inflammatory cytokines such as IL-1β and IL-18 but also reduces the expression and activity of cleaved caspase 1 in lung tissues. Calycosin also plays a protective role in reducing oxidative stress by increasing the levels of SOD and GSH while reducing the concentration of MDA, this being a product of lipid peroxidation involved in oxidative injury [160]. In LPS-induced MLE-12 cells, calycosin up-regulates miR-375-3p expression and is targeted at silencing Rho-associated coiled-coil-containing protein kinase 2 (ROCK2), attenuating apoptosis and inflammation and increasing cell viability with the addition of 3.75, 7.5, 15, 30, or 50 μg/mL calycosin [161]. Calycosin inhibits the assembly of inflammasomes by suppressing interactions between apoptosis-associated speck-like protein containing a CARD (ASC), caspase 1, and NLRP3, enabling it to attenuate inflammation in bone marrow-derived macrophages (BMDMs) [162].

Therefore, calycosin can attenuate sepsis-induced acute lung injury by inhibiting the HMGB1/MyD88/NF-κB pathway and NLRP3 inflammasome activation. The capacity of calycosin to eliminate ROS and modulate NF-κB activity provides us with an additional approach to the treatment of diseases related to inflammation and oxidative stress.

#### 2.7.2. Bacterial, Viral, and Parasitic Infections

Bacteria, viruses and parasites cause different degrees of infections, and around the world, about 5 million people die from bacterial diseases each year [163]. Calycosin has demonstrated its anti-bacterial activity by directly inhibiting mobilized colistin resistance-1 (MCR-1) activity and restoring the anti-bacterial activity of polymyxin B in mcr-1-positive bacterial strains at the concentration of 32 µg/mL, like E. coli DZ2-12R, K. pneumoniae ZJ02 and K. pneumoniae ZJ05 [164]. In BALB/c mice with Respiratory Syncytial Virus (RSV)-induced asthma, calycosn obviously mitigates airway hyperresponsiveness and inflammatory cells’ infiltration at a dose of 0.174 mg/g by enhancing Th1 response and inhibiting Th2/Th17 responses to up-regulate interferon-γ expression for asthma treatment [165]. Angiostrongylus cantonensis (A. cantonensis) are zoonotic nematodes that can cause angiostrongyliasis with central nervous system inflammation and eosinophilic meningitis [166]. In A. cantonensis-induced BALB/c mice angiostrongyliasis, it was found that a co-therapy of calycosin and Albendazole can effectively inhibit inflammation progression in the brain by decreasing the production of inflammatory mediators, such as COX-2, IL-1β and TNF-α, with the intraperitoneal administration of calycosin 30 mg/kg, which also decreases the permeability of the blood brain barrier (BBB) while increasing antioxidant HO-1 expression [167]. Meanwhile, the administration of calycosin avoids the use of anti-bacterial drugs and other corticosteroids, limiting their adverse effects. This suggests that calycosin potentially represents a new therapeutic agent for treating bacterial, viral and parasitic infection-induced diseases.

Based on all the above expositions, Table 1 describes the pharmacological effects and mechanisms of calycosin when used for treating the above inflammatory diseases.

### 2.8. Tumor and Cancer

Studies indicate that long-term chronic inflammation is an important cause of tumorigenesis, including inflammatory responses and cytokines. The number of deaths caused by cancer each year is about 10 million due to the lack of any highly effective treatment plan [168]. Calysoin shows efficient anti-cancer effects, and is involved in inhibiting proliferation, inducing apoptosis and inhibiting migration and invasion. Herein, we describe the anti-cancer mechanisms of calycosin via its three different roles against tumors or cancers.

#### 2.8.1. Apoptosis Induction

In colorectal cancer (CRC), calycosin at the concentration of 50 μM induces cancer cells apoptosis and inhibits invasion by up-regulating and activating SIRT1 and AMPK, followed by inhibiting the Akt/mTOR signaling pathway in human colorectal (HT29) carcinoma cells [169]. In the estrogen receptor β (ERβ)-positive human CRC cell line SW480, calycosin within the concentration range of 10–80 μM up-regulates the expression of ERβ, decreases levels of IGF-1R and active Akt, and down-regulates microRNAs-95 (miR-95), thus promoting cancer apoptosis. Similarly, calycosin within the concentration range of 10–100 μM facilitates ERβ expression, decreases miR-17, and up-regulates PTEN in HCT-116 CRC cells. miR-17 acts similarly to miR-95 but exerts effects by inhibiting PTEN expression [170,171]. Moreover, calycosin has been demonstrated to induce apoptosis in estrogen receptor-positive (ER+) breast cancer by reducing the level of active Akt and its downstream HOX transcript antisense RNA (HOTAIR), which functions as a long non-coding RNA (lncRNA) and is highly expressed in breast cancer, but it is negatively regulated by the PI3K/Akt pathway in MCF-7 cell lines at the effective concentration of 20–100 μM [172]. Regarding osteosarcoma (OS), in human OS MG-63 cells, 0–100 μM calycosin increases the expression of ERβ and then inhibits the activation of the downstream PI3K/Akt pathway to facilitate cancer cell apoptosis, in which ERβ is a tumor suppressor. In the normal osteoblast cell line hFOB1.19, 0–100 μM calycosin had negligible effects on normal cell proliferation according to the MTT assay performed for for 24, 48, and 72 h, indicating its selectivity to cancer cells and low cytotoxicity towards benign cells [173]. By contrast, calycosin within the concentration range of 25–100 μM can induce the apoptosis of ER+ MG-63 human osteosarcoma cells by increasing the expression of proteins related to the PI3K/Akt/mTOR pathway; meanwhile, it has no significant effect on ER-U2-OS cells, indicating its selective action on ER+ cells [174]. In the human osteosarcoma cell line 143B, calycosin similarly suppresses cell proliferation and induces apoptosis by inhibiting the expression of miR-223, which is a short RNA molecule, and this facilitates cell growth and differentiation and decreases the proteins of NF-κB/p65 and IκBα at the dose of 30–120 mg/kg/day in in vivo mouse transplant tumor models and in the concentration range of 60–180 μM in in vitro 143B cell lines [175].

Calycosin within the concentration range of 25–100 μM can increase the apoptosis rate in human ovarian carcinoma SKOV3 cells by up-regulating the ratio of Bax/Bcl-2 and increasing the expressions of caspase proteins, such as caspase-9 and caspase-3, in which Bax is a pro-apoptotic protein, while Bcl-2 is an anti-apoptotic protein. The ratio of Bax/Bcl-2 and the activation of caspase are crucial to apoptosis progression [176]. Similarly, after treatment with 47 μM calycosin in gastric cancer cell AGS, cancer cell apoptosis is promoted by the increased ROS level and activated MAPK/STAT3/NF-κB pathway, resulting in G0/G1 cell cycle arrest and lower cell proliferation. Meanwhile, in gastric cancer cell lines, the inhibitory effect of calycosin is superior to that of cisplatin, while in normal cell lines such as GES-1, IMR-90, L-02, and 293T, the cytotoxicity of calycosin is significantly lower than that of cisplatin, according to the IC_50_ comparison performed using the cell counting kit-8 (CCK-8) assay, showing its selectivity to cancer cells and negligible effect on benign cells [177]. In human papillary thyroid (B-CPAP) cancer cells subjected to 100 μM calycosin treatment, we see that activation of Sestrin2 (SESN2) and the subsequent up-regulation of p-AMPK and inhibition of p-mTOR, promoting cancer cell autophagy and apoptosis [178]. Moreover, calycosin within the concentration range of 1–100 μM promotes cell apoptosis by down-regulating the expression of Bcl-2 and up-regulating the expression of Bax and caspase-3. Meanwhile, G0/G1 phase cell cycle arrest occurs in HepG2 hepatocellular carcinoma cells via the activation of the MAPK, STAT3, NF-κB, and Akt pathways [179].

#### 2.8.2. Migration and Invasion Inhibition

In ER+ breast cancer, on the one hand, calycosin inhibits the metastasis and invasion of cancer cells in the human breast cancer cell lines T47D and MCF-7 within the concentration range of 100–400 μM by suppressing the basic leucine zipper ATF-like transcription factor (BATF)/transforming growth factor β1 (TGFβ1) signaling pathway, helping to inhibit cancer cells’ epithelial–mesenchymal transition (EMT), in the context of which BATF facilitates cell migration and invasion by increasing TGFβ1 expression [180]. On the other hand, in the same cell lines as above, calycosin decreases forkhead box P3 (Foxp3) expression and subsequently down-regulates vascular endothelial growth factor (VEGF) and MMP-9 at high doses to inhibit migration and invasion within the concentration range of 50–150 μM [181]. Furthermore, in the estrogen receptor-negative (ER-) breast cancer cell line MDA-MB-231, 150 μM calycosin reduces the expression of Rab27B and its downstream β-catenin-induced VEGF expression to inhibit cancer cell migration and invasion [182]. Calycosin inhibits cells’ migration and metastasis by suppressing the expression of IκBα/epithelial cell transforming sequence 2 (ECT2) in the human cell line 143B within the concentration range of 60–180 μmol/L and BALB/c nude mice in the dose range of 30–120 mg/kg. ECT2 is an important oncoprotein in the context of the cell cycle, checkpoint and metastasis [183]. In addition, calycosin mainly inhibits glioblastoma migration and invasion in human U87 and U251 cell lines; one pathway is the down-regulation of TGFβ and the inhibition of its induced EMT and MMP activation with calycosin applied in the concentration range of 0–200 μM [184]. The other pathway is the down-regulation of inflammatory chemokine C-X-C chemokine ligand 10 (CXCL10), followed by downstream targets NLRP3, NF-κB, and IL-1β, which are down-regulated within the calycosin concentration range of 100–400 μM. The CCK-8 assay showed calycosin’s greater inhibitory activity against glioma cells, with a high expression of CXCL10 and no significant toxicity to human normal astrocytes, suggesting its selectivity to cancer cells [185]. Moreover, calycosin inhibits the cell viability and invasion of human cervical cancer cell lines SiHa and CaSki in a dose-dependent relationship at 30–50 μM by up-regulating tumor suppressor miR-375, with no significant toxicity to normal cervical epithelial cells Etc1/E6E7 according to the MTT assay and Lactate dehydrogenase (LDH) release assay, suggesting its safety for normal cells [186].

#### 2.8.3. Proliferation and Growth Inhibition

In ER-breast cancer, calycosin can up-regulate the expression of lncRNA WDR7-7 and decrease the G-protein coupled estrogen receptor 30 (GPR30) level in MDA-MB-468 and SKBR3 cell lines at the effective concentration of 4–16 μM, enabling it to inhibit cancer cell growth. WDR7-7 is a tumor suppressor, with negligible effects on normal MCF10A cells according to the CCK-8 and BrdU assays; furthermore, the colony formation assay exhibited that calycosin has no effects on the long-term proliferation ability of normal cells. After daily gavages of 55 mg/kg calycosin in vivo, the growth of MCF-7 and SKBR3 xenograft tumors is significantly inhibited [187]. Moreover, calycosin treatment inhibits the proliferation and metastasis of LUAD A549 cells in the effective concentration range of 20–40 μM via the suppression of the protein kinase C-α (PKC-α)/extracellular signal-regulated kinase 1/2 (ERK1/2) pathway, followed by the decreased expression of MMP-2 and MMP-9 [188]. The anti-proliferation property of calycosin was demonstrated in the nasopharyngeal carcinoma cell lines CNE1 and CNE2 at the effective concentration of 8–50 μM via decreasing the expressions of lncRNA Ewing sarcoma-associated transcript 1 (EWSAT1) and the downstream (tumor necrosis factor receptor-(TNFR)-associated factor 6) TRAF6, p-TAK1, and p-IκBα/p-c-Jun pathways, which are involved in the development of inflammation and tumorigenesis. Meanwhile, the normal proliferation of NP69 cells was not affected by calycosin, as confirmed by the CCK-8 and BrdU assay following the application of 50 μM calycosin for 12, 24, and 48 h [189]. Regarding gastric cancer, in the N-methyl-N′-nitro-N-nitrosoguanidine (MNNG)-induced precancerous lesions of gastric carcinoma (PLGC) rats, calycosin down-regulates levels of NF-κB and DARPP-32, which enact the transformation of intestinal metaplasia and dysplasia, and STAT3, enabling it to protect against gastric injury at doses of 40 and 80 mg/kg [190]. On the other hand, for pancreatic cancer, calycosin plays inhibitory roles in the human pancreatic ductal adenocarcinoma (PDAC) cell lines PANC1 and MIA PaCa-2; it inhibits cell growth and viability in the effective concentration range of 50–100 μM by facilitating S phase cell cycle arrest via the TGF-β-induced activation of the CDK inhibitor p21Waf1/Cip1 [191].

Collectively, calycosin shows its potential anti-cancer activity in many ways, including apoptosis induction, the suppression of migration and invasion, proliferation inhibition, inflammation resistance, and cell cycle arrest. Compared to conventional chemotherapy, there are few severe side effects. Meanwhile, the co-treatment of calycosin and other natural products or chemotherapy drugs may inhibit tumorigenesis and tumor progression more effectively, and thus optimize cancer treatment. More studies are required to develop new and efficient drug designs against different tumors and cancers (Table 2).

## 3. Application Prospects

In this text, we have shown that calycosin is a natural compound classified as an isoflavonoid, and is commonly found in many medicinal herbs, especially the traditional Chinese herb *Radix astragali*. This traditional Chinese medicinal monomer attracts a lot of attention because of its low cytotoxicity and multiple effects [14]. Studies indicate that calycosin holds promising application prospects in treating inflammatory diseases.

Although there are now well-established therapeutic drugs for inflammatory diseases, their adverse effects cannot be ignored. For instance, NSAIDs are associated with gastrointestinal ulceration and mucosal injury. Glucocorticoids predispose patients to opportunistic infections, immunosuppression and osteoporosis, while chemotherapeutic agents frequently induce severe emesis and cardiotoxicity. However, there have been no further in vivo studies conducted to ascertain calycosin’s harmfulness so far. As a herbal medication, calycosin has demonstrated many medical benefits and is virtually non-toxic, with minor side effects shown in recent studies, corroborating its efficacy as a treatment and giving hope for the treatment of many diseases [192].

Moreover, more and more interactions between calycosin and other biochemicals are being reported, showing more potentially therapeutic effects compared to the single compound [193]. The cotreatment of albendazole and calycosin can significantly attenuate Angiostrongylus cantonensis-induced parasitic meningitis and inhibit the release of inflammatory mediators such as TNF-α and IL-1β, showing better effects than treatments with a single medicine [167]. Calycosin exhibits a coordination effect in the Danggui Buxue Tang (DBT), enabling it to exert erythropoietic and osteogenic abilities, as shown via comparison with a calycosin-depleted DBT decoction [194]. Interestingly, the anti-inflammatory ability of calycosin is more potent than its glucoside “(2S,3R,4S,5S,6R)-2-[(7-Hydroxy-3-(4-hydroxyphenyl)-4-oxo-4H-chromen-8-yl)oxy]-6-(hydroxymethyl)oxane-3,4,5-triol”, indicated via evaluations of the production of IL-12 p40 by LPS-stimulated bone marrow-derived dendritic cells (BMDCs) [195]. Additionally, calycosin was proposed as the substrate of most CYP450 subunits, like CYP1A2, CYP2C9, and CYP2D6, and can be easily eliminated from the body after its intended medical use has been exhausted, according to SOHEL et al.’s research [196]. Meanwhile, drug–drug interactions must be noted in clinical contexts. Drug-metabolizing enzymes (DMEs) and efflux transporters (ETs) are crucial to the metabolism, elimination and detoxification of drugs, but are affected by their substrates. In particular, DME CYP3A4 can easily cause drug–drug interactions, leading to large changes in drug plasma concentrations, and even causing adverse effects in the body. This study has demonstrated that calycosin may up-regulate the expressions of some DMEs, such as CYP3A4, and certain ETs, thus affecting drug–drug interactions. Therefore, moderate monitoring is necessary in the context of co-medication with other drugs [197]. Moreover, nano-delivery platforms can be combined with calycosin to transport it to its precise site of action, amplifying its potential therapeutic efficacy while minimizing off-target exposure [196].

Despite the promising in vitro activities summarized above, calycosin exhibits the common flavonoid drawbacks of low oral bioavailability due to poor intestinal absorption and extensive first-pass metabolism, primarily via glucuronidation and sulfation. Several isoflavonoids, including calycosin, have been flagged as potential PAINS due to their tendency to form reactive quinones, chelate metals, or aggregate non-specifically in vitro [198]. These properties may lead to false-positive results in screening assays. Future studies should employ PAINS-filtered assays, include appropriate negative controls, and validate in vitro findings using pharmacologically relevant concentrations in vivo [199]. In addition, Yu, S. et al. found that calycosin exhibits inhibitory efficacy against human T follicular helper (Tfh) differentiation by targeting BATF, indicating its potential for clinical translation when used against autoimmune diseases like Sjögren’s [200]. Given the PAINS problem and the absence of formal pre-clinical safety data, calycosin should presently be regarded only as a tool compound. Substantial structural refinement would be required before planning any realistic clinical development.

In summary, although current studies have focused on animal experiments, many results suggest a potential benefit of calycosin in the treatment of inflammatory diseases. Its anti-inflammatory, antioxidant, multi-targeted anticancer, and immune-modulatory properties, as well as its synergistic effects with other drugs, provide researchers with new directions related to the management of most inflammatory diseases.

## 4. Conclusions

A summary of the available studies shows that calycosin plays effective roles in many inflammatory diseases. First, calycosin has shown its anti-inflammatory properties mainly by modulating the NF-κB and MAPK signaling pathways, thereby reducing the production of pro-inflammatory cytokines. This characteristic enables calycosin to alleviate inflammatory conditions such as kidney damage, acute liver failure and acute pancreatitis. Furthermore, calycosin demonstrates antioxidant effects by eliminating reactive oxygen species (ROS) and enhancing the activities of the antioxidant enzyme, thereby reducing oxidative stress and preventing the tissue damage is causes [201]. These two mechanisms not only enhance the potential therapeutic prospects of calycosin, but also highlight its variability in treating diseases characterized by inflammation and oxidative damage. Meanwhile, studies have shown that calycosin can inhibit the activation of inflammasomes such as NLRP3, thereby attenuating inflammatory responses [2]. The abnormal activation of the NLRP3 inflammasome has been confirmed in inflammatory diseases such as rheumatoid arthritis. This indicates that the regulatory role of calycosin could offer novel treatment strategies for these diseases, associated with inflammatory responses and oxidative damage.

Although calycosin shows promising therapeutic potential for use against multiple diseases, most studies thereof have been conducted using animal models and cell cultures. Its results in humans, its pharmacokinetic profile and its drug–drug interactions have not yet been clarified. Further research focusing on absorption, distribution, metabolism, excretion, and toxicity should be conducted using online bioinformatic tools, including the QuickPro module and admetSAR, to provide reliable data for clinical use. Similarly, many clinical trials and clinical data are needed to support these points. Nevertheless, as a kind of traditional Chinese medicine, calycosin appears to cause very few adverse effects in patients compared with other artificially synthesized chemical drugs. Undoubtedly, calycosin is becoming a popular topic and represents a novel direction for scientific research.

## Figures and Tables

**Table 1:** Therapeutic mechanisms of calycosin in the context of inflammatory diseases.

Inflammatory Diseases	Cell Lines or Animal Models	Dosage	Specific Mechanisms	Application	References
Atherosclerosis	ApoE-deficient mice	60 mg/kg/day	Up-regulate expression of KLF2 and inhibit expression of MLKL, decrease inflammatory cascade reactions	In vivo	[26]
	Male C57BL/6 mice	50 mg/kg/day	Increase adiponectin level, activate AMPK phosphorylation and promote eNOS to product NO	In vivo	[27]
	Rat thoracic aortic smooth muscle cell line A7r5	5–20 µM	Activate AMPK/mTOR pathway to induce autophagy and SNARE complex-mediated autophagosome-lysosome fusion	In vitro	[29]
Heart failure	TGFβ-induced cardiac fibroblast model	5 µM	Decrease α-SMA expression and reduce p-STAT3 and MMP-9 levels	In vitro	[35]
	Male C57/BL6 mice	50 mg/kg/day	Inhibit TGFBR1 pathway and down-regulate intracellular signal transducers Smad2/3	In vivo	[36]
	Left anterior descending (LAD) artery ligation-induced heart failure rat model	5 µM	Increase expression of PI3K and phosphorylated Akt	In vivo	[34]
	Adult zebrafish doxorubicin-induced cardiotoxicity model	5 µmol/L	Increase the expression of Atg7 to promote autophagy recovery	In vivo	[37]
	Doxorubicin stimulated H9c2 cells	0–20 μM	Inhibit NLRP3 inflammasome-induced pyroptosis	In vitro	[38]
		50–200 μM	Up-regulate Sirt 1 expression but down-regulate NLRP3 expression	In vitro	[39]
	Triptolide-induced cardiotoxicity H9c2 cardiomyocytes	100 μM	Promote formation of PGC-1α/NRF1 complex	In vitro	[41]
Myocardial infarction	Isoproterenol-induced myocardial infarction mice model	40 mg/kg/day	Induce LTB4DH expression and reduce neutrophil infiltration	In vivo	[48]
	Neonatal rat cardiomyocytes and C57BL/6 mouse myocardial infarction model	2.5–10 µM 10–20 mg/kg/day	Increase ALDH2 activity, down-regulate Bax expression and up-regulate Bcl-2 expression	In vitro and vivo	[45]
	H9C2 myocardial cells	5–20 µM	Increase ERα/β expression and enhance PI3K/Akt phosphorylation	In vitro	[46]
	Adult male Sprague Dawley rats	4 mg/kg	Activate VEGF expression and increase CD31 expression to promote angiogenesis	In vivo	[47]
Hypertension	Isolated male Sprague Dawley rat thoracic aortic rings	30 μmol/L	Inhibit VOC and ROC, respectively, in KCl and phenylephrine induced contraction	In vivo	[54]
	Human umbilical vein endothelial cells	1–100 µM	Activate endothelial NOS/neural NOS-dependent NO production and BK_Ca_, enhance endothelium hyperpolarization	In vitro	[55]
Osteoarthritis	IL-1β treated chondrocytes	100–400 µM	Inhibit PI3K/AKT and NF-κB pathway	In vitro	[64]
	Human primary chondrocytes stimulated with IL-1β	1–10 µM	Improve cartilage formation via FoxO1	In vitro	[65]
	Anterior cruciate ligament transection mouse model Chondrocytes ADTC5 cells	50 mg/kg 16–64 μM	Increase cartilage synthesis biomarkers Col-2 and Sox-9, decrease COX-2 and EGFR, improve balance of cartilage synthesis and degradation	In vitro and vivo	[66]
Gouty arthritis	Mouse model induced by MSU Peripheral blood mono-nuclear cells (PBMCs) and THP-1 cells	50 mg/kg 10 μM	Inhibit activation of the NF-κB pathway and modulate p62-Keap 1 pathway	In vitro and vivo	[70]
Rheumatoid arthritis	Rheumatoid arthritis synovial fibroblasts	10–100 μM	Down-regulate expression of proinflammatory cytokines by activating p62/Nrf2-linked heme oxygenase 1	In vitro	[73]
	Collagen-induced arthritis mouse model	1 mg/kg	Inhibit activation of inflammatory pathway modulators JNK, IKKα/β, and p65	In vivo	[74]
	None	None	Bind to and inhibit IL-6R with high affinity	None	[75]
Acute pancreatitis	Balb/C mice models	20 or 50 mg/kg	Inhibit expression of NF-κB/p65 and phosphorylate IκBα and p38 MAPK	In vivo	[80]
			Decrease MPO level and increase SOD activity		
Acute liver failure	LPS-induced human liver epithelial cell line (L02) cells CCl4-induced C57BL/6 mice liver injury	12.5–50 mg/kg	Increase FXR target gene FoxM1B and SHP expression and STAT3 phosphorylation	In vivo	[85]
Non-alcoholic fatty liver disease	C57BL/6J male mice fed with high fat diet-induced NAFLD model	50 mg/kg	Facilitate FXR activation, regulate Shp/Pepck/G-6-pase pathway, improve glucose and lipid metabolism, and glucose transport	In vivo	[91]
	NASH mice model	12.5–50 mg/kg	Increase fatty acid β-oxidation, mitigate liver fibrosis	In vivo	[92]
	Carbon tetrachlorid-induced liver fibrosis mice HSC-T6 cells LX-2 cells	20–80 mg/kg 25–200 μM 25–200 μM	Increase ERβ expression and activate subsequent JAK2-STAT3 to inhibit hepatic stellate cells activation and collagen deposition	In vivo and vitro	[93,94,95,96]
Diabetes mellitus	Normal rat hepatocyte cell line (BRL-3A) cultured by high glucose	1 × 10^−7^ M	Reverse Glut-1 expression, reduce AGE receptor expression and directly bind to AGEs	In vitro	[102]
		IC_50_ = 6.84 ± 1.58 µM	Inhibit α-glucosidase, inhibit glucose uptake into blood	In vitro	[104]
	Human umbilical vein endothelial cells	10^−4^ M	Inhibit AGEs-RAGE ligation and downstream NF-κB inflammatory pathway and increase Bcl-2 expression, decreasing Bad/Bax expression	In vitro	[103]
	Pregnant db/+ diabetic mice	15 and 30 mg/kg	Suppress RNF38 expression, inhibit STAT3 activation, and increase SHP-1 expression	In vivo	[105]
Colitis	Dextran sulfate sodium-induced colitis mice	25 and 50 mg/kg	Inhibit NF-κB and phosphorylation of IKKα/β, IκBα and p65	In vivo	[109]
			Increase concentration of GSH and SOD to alleviate abnormal redox reactions		
	Human intestinal fibroblasts (CCD-18Co) cells	12.5–50 μmol/L	Inhibit TGF-β/Smad pathway	In vitro	[110]
Diabetes-induced renal inflammation	The db/db mice and mouse tubular epithelial cells	10 mg/(kg·d) 10 μM	Inhibit phosphorylation of IκBα and NF-κB p65	In vivo and vitro	[115]
	Mouse tubular epithelial cells	5–80 μM	Inhibit ferroptosis iron-dependent cell death	In vitro	[116]
	High fat diet-fed/STZ injected rats	5 mg/kg	Inhibit IL-33/ST2 axis, substantially activate NF-κB inflammatory pathway and TGF-β/Smad pathway, and activate Nrf2/ARE pathway	In vivo	[117]
Renal ischemia/reperfusion injury	Mice with renal ischemia/reperfusion injury	5–20 mg/kg	Up-regulate expression of PPARγ and suppress EGR1	In vivo	[123]
Chronic prostatitis	Rats with chronic prostatitis	10–30 mg/kg	Inhibit activation of p38MAPK/NF-κB signaling pathway	In vivo	[126]
Intracerebral hemorrhage-induced brain damage	Collagenase type VII-induced intracerebral hemorrhage mouse model	50 mg/kg	Block activation of NLRP3 inflammasome and classical NF-κB pathway	In vivo	[130]
Cerebral ischemia injury	Oxygen-glucose deprivation/reoxygenation (OGD/R) model of rat microglia	1–4 μM	Decrease HMGB1/TLR4/NF-κB signaling pathway	In vitro	[134]
	Rat middle cerebral artery occlusion/reperfusion (MCAO/R) model and PC12 cells	30 mg/kg 60 μM	Inhibit autophagy via STAT3/FOXO3a signaling pathway and ACSL4 dependent ferroptosis	In vivo and vitro	[131,135]
	Middle cerebral artery occlusion (MCAO) rats	5–20 mg/kg	Increase expression of autophagy-related protein p62 and NBR1 and anti-apoptotic Bcl-2, decrease TNF-α expression	In vivo	[136]
		5–20 mg/kg	Reduce RASD1 expression, up-regulate ER-α, miR-375 and Bcl-2	In vivo	[138]
		0.44 mg/kg	Activate S1P/S1PR1/PI3K/Akt pathway	In vivo	[139]
		30 mg/kg	Increase BDNF and TrkB expression in brain to switch the TNF-α containing microglia from the activated state to the resting state	In vivo	[137]
		5–20 mg/kg	Inhibit calpain activation and increase TRPC6 and CREB expression	In vivo	[141]
	Rat brain astrocytes	0–100 μM	Activate Akt, promote phosphorylation of Nrf2 and downstream HO-1 and SOD activation to limit H2O2-induced ROS production	In vitro	[143]
Allergic dermatitis	Balb/C mice and HaCaT cell	2–50 mg/kg 10 μmol/L	Down-regulate expression of HIF-1α to repair epithelial tight junctions	In vivo and vitro	[144,146]
	House dust mite (HDM)-induced allergic asthma mouse model and TNF-α and Poly (I:C) co-stimulated human bronchial epithelial cell line	10 mg/kg 10 μM	Increase occludin expression, improve E-cadherin distribution, and inhibit TSLP production	In vivo and vitro	[147]
Atopic dermatitis	Initial stage of AD model and HaCaT cells	0.4–10 mg/kg 0.1–10 μM	Inhibit TLR4 mediated NF-κB signaling pathway	In vivo and vitro	[151]
	Calcipotriol-induced mouse model HaCaT cells	1–5 mg/mL 2–10 μM	Promote Treg cells differentiation but inhibit Th17 cells	In vivo and vitro	[154]
Sepsis-induced acute lung injury	The cecal ligation and puncture (CLP)-treated young rats	50 mg/kg	Inhibit the HMGB1/MyD88/NF-κB pathway and activate NLRP3 inflammasome	In vivo	[158]
		12.5–50 mg/kg	inhibit mitochondrial ROS mediated inflammasome activation	In vivo	[160]
	LPS-induced MLE-12 cells	3.75–50 μg/ml	Up-regulate miR-375-3p expression and silence ROCK2 to attenuate inflammation and increase cell viability	In vitro	[161]
Bacterial, viral, and parasitic infections	Mcr-1-positive bacterial strains	32 µg/ml	Inhibit MCR-1 activity and restore the anti-bacterial activity of polymyxin B	In vitro	[164]
	Respiratory Syncytial Virus (RSV)-induced asthma-exacerbated BALB/c mice	0.174 mg/g	Enhance Th1 response and inhibit Th2/Th17 responses to up-regulate interferon-γ expression	In vivo	[165]
	A. cantonensis-induced angiostrongyliasis BALB/c mice	30 mg/kg	Facilitate antioxidant HO-1 expression and inhibit NF-κB pathway activation	In vivo	[167]

ACSL4, acyl-CoA synthetase long-chain family member 4; AGEs, advanced glycation end products; Akt, serine/threonine kinase; ALDH2, aldehyde dehydrogenase 2; AMPK, adenosine 5′-monophosphate (AMP)-activated protein kinase; ApoE^−/−^, apolipoprotein E gene-deficient; ARE, antioxidant response element; ASC, apoptosis-associated speck-like protein containing a CARD; BDNF, brain-derived neurotrophic factor; BKCa, large-conductance calcium activated potassium channels; BMDMs, bone marrow-derived macrophages; CD31, cluster of differentiation 31; CM, conditioned media; Col-2, type II collagen; COX-2, cyclooxygenase-2; CREB, cAMP-response element binding protein; db/+, C57BL/KsJ-Lep; db/db, C57BL/KsJ; Drp1, dynamin-related protein 1; DSS, dextran sulfate sodium; EGFR, epidermal growth factor receptor; EGR1, early growth response 1; eNOS, endothelial nitric oxide synthase; ERα/β, estrogen receptor α/β; FoxO1, forkhead box O1; FXR, farnesoid X receptor; G-6-pase, glucose-6-phosphatase; Glut-1, glucose transporter-1; Glut-4, glucose transporter 4; GSH, glutathione; HIF-1α, hypoxia-inducible factor-1α; HMGB1, high mobility group protein 1; HO-1, heme oxygenase-1; IKK, IκB kinase; IL, interleukin; IL-6R, interleukin-6 receptor; JAK2, Janus kinase 2; JNK, c-Jun N-terminal kinase; Keap1, Kelch-like ECH-associated protein 1; KLF2, krüppel-like factor 2; LPS, lipopolysaccharide; LTB4DH, Leukotriene B4 12-hydroxydehydrogenase; MAPK, mitogen-activated protein kinases; MCR-1, mobilized colistin resistance-1; MDA, malondialdehyde; miR, microRNAs; MLKL, mixed-lineage kinase domain-like protein; MMP-9, matrix metalloproteinase-9; MMPs, matrix metalloproteinases; MPO, myeloperoxidase; MSU, monosodium urate; mTOR, mammalian target of rapamycin; MyD88, myeloid differentiation factor 88; NAFLD, non-alcoholic fatty liver disease; NASH, nonalcoholic steatohepatitis; NBR1, neighbor of BRCA1 gene 1; NF-κB, nuclear factor kappa-B; NLRP3, NOD-like receptor 3; NO, nitric oxide; NQO1, NAD(P)H dehydrogenase quinone 1; NRF1, nuclear respiratory factor-1; Nrf2, nuclear factor-erythroid 2-related factor 2; Pepck, Phosphoenolpyruvate carboxykinase; PGC-1α, peroxisome proliferator-activated receptor gamma coactivator-1 alpha; PI3K, phosphatidylinositol three kinase; PPARγ, peroxisome proliferator-activated receptor γ; RASD1, Ras-related protein 1; RNF38, ring finger protein 38; ROC, receptor-operated calcium channels; ROCK2, Rho-associated coiled-coil-containing protein kinase 2; ROS, reactive oxygen species; S1P, Sphingosine1-phosphate; S1PR1, Sphingosine-1-phosphate receptor 1; Shp, small heterodimer partner; SHP-1, SH2-containing protein tyrosine phosphatase 1; Sirt1, sirtuin 1; SNARE, soluble N-ethylmaleimide-sensitive factor attachment protein receptor; SOD, superoxide dismutase; Sox-9, SRY-Box Transcription Factor 9; ST2, growth stimulation-expressed gene 2; STAT3, signal transducer and activator of transcription 3; TGFBR1, transforming growth factor-beta receptor 1; TGF, transforming growth factor; Th, T helper; TLR, toll-like receptor; TNF-α, tumor necrosis factor α; Treg, Regulatory T; TrkB, tropomyosin-related kinase B; TRPC6, transient receptor potential canonical 6; VEGF, vascular endothelial growth factor; VOC, voltage-operated calcium channels; α-SMA, α-smooth muscle actin.

**Table 2:** Anti-cancer and anti-tumor mechanisms of calycosin.

Category	Cancer Cell Lines or Animal Models	Specific Mechanisms	Dosage	References
Apoptosis induction	Human colorectal (HT29) carcinoma cells	Activate Sirt1 and AMPK, inhibit Akt/mTOR signaling pathway	50 μM	[169]
	Human colorectal cancer cell lines SW480	Up-regulate ERβ, decrease IGF-1R and Akt, down-regulate miR-95	10–80 μM	[170]
	HCT-116 CRC cells	Increase ERβ expression, decrease miR-17, and up-regulate PTEN	10–100 μM	[171]
	Human breast cancer cell lines MCF-7	Deactivate HOTAIR/p-Akt signaling pathway	20–100 μM	[172]
	Human osteosarcoma MG-63 cells	Increase ERβ expression, inhibit activation of PI3K/Akt pathway	100 μM	[173]
	ER-positive MG-63 human osteosarcoma cells	Increase protein expression of PI3K/Akt/mTOR pathway	25–100 μM	[174]
	Human osteosarcoma cell line 143B	Inhibit miR-223 expression, decrease NF-κB/p65 and IκBα	60–180 μM	[175]
	Human ovarian carcinoma SKOV3 cells	Up-regulate ratio of Bax/Bcl-2 and increase expression of caspase protein	25–100 μM	[176]
	Gastric cancer cells AGS	Increase ROS level and MAPK/STAT3/NF-κB pathway	47 μM	[177]
	Human papillary thyroid (B-CPAP) cancer cells	Activate SESN2 and up-regulate p-AMPK, inhibit p-mTOR	100 μM	[178]
	HepG2 hepatocellular carcinoma cells	Down-regulate Bcl-2, up-regulate Bax and caspase-3	1–100 μM	[179]
Migration and invasion inhibition	Human breast cancer cell lines T47D and MCF-7	Suppress BATF/TGFβ1 signaling pathway	100–400 μM	[180]
	Human breast cancer cell lines T47D and MCF-7	Decrease Foxp3 expression and down-regulate VEGF and MMP-9	50–150 μM	[181]
	ER- breast cancer cell line MDA-MB-231	Reduce Rab27B expression and β-catenin-induced VEGF	150 μM	[182]
	Human cell line of 143B and BALB/c nude mice	Suppress IκBα/ECT2 expression and reduce IL-6 and MMPs	60–180 μmol/L 30–120 mg/kg	[183]
	Human U87 and U251 cell lines	Down-regulate TGFβ and inhibit activation of EMT and MMP	0–200 μM	[184]
	Human U87 and U251 cell lines	Down-regulate inflammatory chemokine CXCL10	100–400 μM	[185]
	Human cervical cancer cell lines SiHa and CaSki	Decrease tumor suppressor miR-375	30–50 μM	[186]
Proliferation and growth inhibition	MDA-MB-468 and SKBR3 cell lines	Up-regulate lncRNA WDR7-7 and decrease GPR30 level	4–16 μM	[187]
	LUAD cells A549	Suppress PKC-α/ERK1/2 pathway and decrease MMP expression	20–40 μM	[188]
	Nasopharyngeal carcinoma cell lines CNE1 and CNE2	Decrease expression of lncRNA EWSAT1 and TRAF6	8–50 μM	[189]
	MNNG-induced PLGC rats	Down-regulate the levels of NF-κB, DARPP-32, and STAT3	40 and 80 mg/kg	[190]
	PANC1 and PaCa-2 cell lines	TGF-β induced the activation of the CDK inhibitor p21Waf1/Cip1	50–100 μM	[191]

Akt, serine/threonine kinase; AMPK, adenosine 5′-monophosphate (AMP)-activated protein kinase; BATF, basic leucine zipper ATF-like transcription factor; CDK, cyclin-dependent kinases; CRC, colorectal cancer; CXCL10, chemokine C-X-C, chemokine ligand 10; DARPP-32, dopamine- and cAMP-regulated phosphoprotein, Mr 32 kDa; ECT2, epithelial cell transforming sequence 2; EMT, epithelial–mesenchymal transition; ERK1/2, extracellular signal-regulated kinase 1/2; ERβ, estrogen receptor β; EWSAT1, Ewing sarcoma-associated transcript 1; Foxp3, forkhead box P3; GPD1L, glycerol-3-phosphate dehydrogenase 1 like; GPR30, G-protein coupled estrogen receptor 30; HIF-1α, hypoxia-inducible factor-1α; HOTAIR, HOX transcript antisense RNA; IGF-1R, insulin-like growth factor 1 receptor; lncRNA, long non-coding RNA; MAPK, mitogen-activated protein kinases; miR, microRNAs; MMP-9, matrix metalloproteinase-9; MMPs, matrix metalloproteinases; MNNG, N-methyl-N′-nitro-N-nitrosoguanidine; mTOR, mammalian target of rapamycin; NF-κB, nuclear factor kappa-B; PI3K, phosphatidylinositol three kinase; PKC α, protein kinase C-α; PLGC, precancerous lesions of gastric carcinoma; PTEN, phosphatase and tensin homolog; Rab27B, member RAS oncogene family; SESN2, sestrin2; Sirt1, sirtuin 1; STAT3, signal transducer and activator of transcription 3; TGF, transforming growth factor; TRAF6, tumor necrosis factor receptor-associated factor 6; VEGF, vascular endothelial growth factor.

## Data Availability

No new data were created or analyzed in this study.

## Abbreviations

ACSL4	Acyl-CoA synthetase long-chain family member 4
AGEs	Advanced glycation end products
Akt	Serine/threonine kinase
ALDH2	Aldehyde dehydrogenase 2
AMPK	Adenosine 5′-monophosphate (AMP)-activated protein kinase
ApoE^−/−^	Apolipoprotein E gene-deficient
ARE	Antioxidant response element
ASC	Apoptosis-associated speck-like protein containing a CARD
BATF	Basic leucine zipper ATF-like transcription factor
BDNF	Brain-derived neurotrophic factor
BK_Ca_	Large-conductance calcium activated potassium channels
BMDMs	Bone marrow-derived macrophages
CA	Calycosin
CD31	Cluster of differentiation 31
CDK	Cyclin-dependent kinase
CM	Conditioned media
Col-2	Type II collagen
COX-2	Cyclooxygenase-2
CRC	Colorectal cancer
CREB	cAMP-response element binding protein
CXCL10	Chemokine C-X-C chemokine ligand 10
DARPP-32	Dopamine- and cAMP-regulated phosphoprotein Mr 32 kDa
db/+	C57BL/KsJ-Lep
db/db	C57BL/KsJ
Drp1	Dynamin-related protein 1
DSS	Dextran sulfate sodium
ECT2	Epithelial cell transforming sequence 2
EGFR	Epidermal growth factor receptor
EGR1	Early growth response 1
EMT	Epithelial-mesenchymal transition
eNOS	Endothelial nitric oxide synthase
ERK1/2	Extracellular signal-regulated kinase 1/2
ERα/β	Estrogen receptor α/β
ERβ	Estrogen receptor β
EWSAT1	Ewing sarcoma-associated transcript 1
FoxO1	Forkhead box O1
Foxp3	Forkhead box P3
FXR	Farnesoid X receptor
G-6-pase	Glucose-6-phosphatase
Glut-1	Glucose transporter-1
Glut-4	Glucose transporter-4
GPD1L	Glycerol-3-phosphate dehydrogenase 1 like
GPR30	G-protein coupled estrogen receptor 30
GSH	Glutathione
HIF-1α	Hypoxia-inducible factor-1α
HMGB1	High mobility group protein 1
HO-1	Heme oxygenase-1
HOTAIR	HOX transcript antisense RNA
IGF-1R	Insulin-like growth factor 1 receptor
IKK	IκB kinase
IL	Interleukin
IL-6R	Interleukin-6 receptor
JAK2	Janus kinase 2
JNK	c-Jun N-terminal kinase
Keap1	Kelch-like ECH-associated protein 1
KLF2	Krüppel-like factor 2
lncRNA	Long non-coding RNA
LPS	Lipopolysaccharide
LTB4DH	Leukotriene B4 12-hydroxydehydrogenase
MAPK	Mitogen-activated protein kinases
MCR-1	Mobilized colistin resistance-1
MDA	Malondialdehyde
miR	MicroRNA
MLKL	Mixed lineage kinase domain-like protein
MMP-9	Matrix metalloproteinase-9
MMPs	Matrix metalloproteinases
MNNG	N-methyl-N′-nitro-N-nitrosoguanidine
MPO	Myeloperoxidase
MSU	Monosodium urate
mTOR	Mammalian target of rapamycin
MyD88	Myeloid differentiation factor 88
NAFLD	Non-alcoholic fatty liver disease
NASH	Nonalcoholic steatohepatitis
NBR1	Neighbor of BRCA1 gene 1
NF-κB	Nuclear factor kappa-B
NLRP3	NOD-like receptor 3
NO	Nitric oxide
NQO1	NAD(P)H dehydrogenase quinone 1
NRF1	Nuclear respiratory factor-1
Nrf2	Nuclear factor-erythroid 2-related factor 2
Pepck	Phosphoenolpyruvate carboxykinase
PGC-1α	Peroxisome proliferator-activated receptor gamma coactivator-1 alpha
PI3K	Phosphatidylinositol three kinase
PKC-α	Protein kinase C-α
PLGC	Precancerous lesions of gastric carcinoma
PPARγ	Peroxisome proliferator-activated receptor γ
PTEN	Phosphatase and tensin homolog
RA	*Radix astragali*
Rab27B	Member RAS oncogene family
RASD1	Ras-related protein 1
RNF38	Ring finger protein 38
ROC	Receptor-operated calcium channel
ROCK2	Rho-associated coiled-coil-containing protein kinase 2
ROS	Reactive oxygen species
S1P	Sphingosine1-phosphate
S1PR1	Sphingosine-1-phosphate receptor 1
SESN2	Sestrin2
Shp	Small heterodimer partner
SHP-1	SH2-containing protein tyrosine phosphatase 1
Sirt1	Sirtuin 1
SNARE	Soluble N-ethylmaleimide-sensitive factor attachment protein receptor
SOD	Superoxide dismutase
Sox-9	SRY-Box Transcription Factor 9
ST2	Growth stimulation expressed gene 2
STAT3	Signal transducer and activator of transcription 3
TGF	Transforming growth factor
TGFBR1	Transforming growth factor-beta receptor 1
Th	T helper
TLR	Toll-like receptor
TNF-α	Tumor necrosis factor α
TRAF6	Tumor necrosis factor receptor-associated factor 6
Treg	Regulatory T
TrkB	Tropomyosin-related kinase B
TRPC6	Transient receptor potential canonical 6
VEGF	Vascular endothelial growth factor
VOC	Voltage-operated calcium channel
α-SMA	α-smooth muscle actin
